# Respiratory Health – Exposure Measurements and Modeling in the Fragrance and Flavour Industry

**DOI:** 10.1371/journal.pone.0148769

**Published:** 2016-02-10

**Authors:** Eric Angelini, Gerard Camerini, Malick Diop, Patrice Roche, Thomas Rodi, Christine Schippa, Thierry Thomas

**Affiliations:** V. Mane Fils Sa, Le-Bar-Sur-Loup, France; University of Alabama at Birmingham, UNITED STATES

## Abstract

Although the flavor and fragrance industry is about 150 years old, the use of synthetic materials started more than 100 years ago, and the awareness of the respiratory hazard presented by some flavoring substances emerged only recently. In 2001, the US National Institute of Occupational Safety and Health (NIOSH) identified for the first time inhalation exposure to flavoring substances in the workplace as a possible occupational hazard. As a consequence, manufacturers must comply with a variety of workplace safety requirements, and management has to ensure the improvement of health and safety of the employees exposed to hazardous volatile organic compounds. In this sensitive context, MANE opened its facilities to an intensive measuring campaign with the objective to better estimate the real level of hazardous respiratory exposure of workers. In this study, exposure to 27 hazardous volatile substances were measured during several types of handling operations (weighing-mixing, packaging, reconditioning-transferring), 430 measurement results were generated, and were exploited to propose an improved model derived from the well-known ECETOC-TRA model. The quantification of volatile substances in the working atmosphere involved three main steps: adsorption of the chemicals on a solid support, thermal desorption, followed by analysis by gas chromatography-mass spectrometry. Our approach was to examine experimental measures done in various manufacturing workplaces and to define correction factors to reflect more accurately working conditions and habits. Four correction factors were adjusted in the ECETOC-TRA to integrate important exposure variation factors: exposure duration, percentage of the substance in the composition, presence of collective protective equipment and wearing of personal protective equipment. Verification of the validity of the model is based on the comparison of the values obtained after adaptation of the ECETOC-TRA model, according to various exposure scenarios, with the experimental values measured under real conditions. After examination of the predicted results, 98% of the values obtained with the proposed new model were above the experimental values measured in real conditions. This must be compared with the results of the classical ECETOC-TRA system, which generates only 37% of overestimated values. As the values generated by the new model intended to help decision-makers of the industry to implement adapted protective action and information, and considering the high variability of the working environments, it was of the utmost importance to us not to underestimate the exposure level. The proposed correction factors have been designed to achieve this goal. We wish to propose the present method as an improved monitoring tool to improve respiratory health and safety in the flavor and fragrance manufacturing facilities.

## Introduction

Over 3,000 chemically-defined flavoring substances and natural flavoring complexes are available globally to formulate flavors. The vast majority of these materials have chemical and physical characteristics that would make it highly unlikely that they would pose a risk of respiratory injury in the workplace. Most of the materials are not very volatile and do not have a significant degree of reactivity. However, some low molecular weight chemically-defined flavoring substances may have sufficient volatility and possibly reactivity, to pose a risk of respiratory injury when improperly handled [1].

The respiratory (nasal cavity, bronchi, bronchioles, etc.) and cutaneous (skin) routes are the principal exposure pathways. It should be specified that the oral route is almost always accidental and is not taken into account in the table of occupational diseases. Classically two types of toxicity are distinguished: acute toxicity and chronic toxicity. The first one results from an exposure to a high dose over a short period of time. The second one is the consequence of repeated exposure to lower doses, which may develop into various pathologies such as cancer, asthma, reprotoxicity or developmental toxicity (effects on offspring).

To help manufacturers identify the hazard related to a substance, and then prevent the above mentioned poisoning, technical agencies are responsible for issuing toxicological data sheets for each substance produced in order to enable company regulatory departments to establish reliable safety data sheets for their products. In France, the INRS (National Institute for Occupational Research and Safety) is responsible for this. Furthermore the REACH (Registration, Evaluation, Authorization of CHemicals) regulation provides a thorough European inventory of chemicals, with an evaluation or re-evaluation of those which are produced or imported above one ton a year in Europe.

Given that operator exposure to substances or chemical preparations is likely to interfere with their health, an international regulatory framework enables harmonization of the evaluation and management of the chemical risk through the GHS (Global Harmonized System). In Europe, the regulatory framework is set by the EEC Directive No. 89–391 dated June 12th, 1989 relating to the implementation of measures promoting the improvement of health and safety conditions in the workplace [2]. Transposition of this directive into French law has established employer obligations concerning risk assessment for activities likely to present an exposure to hazardous chemicals. This assessment must be renewed periodically taking into account:

- intrinsic hazardous properties of chemicals present in the workplace
- information in the safety data sheets delivered with the products by the suppliers
- additional toxicological information which could be necessary
- nature, degree of exposure and duration
- conditions of use of the substances including their frequency and the volumes handled
- OELVs (Occupational Exposure Limit Values) and BLVs (Biological Limit Values) established by government agencies: INRS in France, OSHA (Occupational Safety and Health Administration) in the USA, SCOEL (Scientific Committee on Occupational Exposure Limits) in Europe,
- effect of preventive measures,
- conclusions provided by the occupational health physician concerning health monitoring and safety of workers within the Company.

As this specifically involves the prevention of chemical risk through inhalation, as soon as the assessment reveals a health risk for the workers, an exposure control [3–6] is carried out in order to check that the regulatory occupational exposure limit values are respected.

The present paper deals only with respiratory risks induced by the presence of vapors in air, and does not report the airborne aerosols and particulates issue, although we know that monitoring of particles have a significant effect on air quality and on the health of individuals. Employees within the flavoring production industry have complex respiratory exposures in terms of the number of different chemicals used and of the high variability of the exposure depending upon the specific work assignment. Although there are thousands of flavoring compounds in use, few have occupational exposure limits. Due to the complex mixed exposures within the industry and the absence of inhalation toxicology data for most chemicals, engineering controls are a matter of utmost importance to the global flavor and fragrance manufacturing industry, in order to maintain workplaces as safe and healthy as possible. Currently, there is no model or standard guidance for engineering controls for flavoring processes and, as a result, a wide range of systems have been observed, many with marginal effectiveness.

At the start of the project, we had in mind to focus on diacetyl; a choice justified by the health risk that it could represent; problem largely publicized in the press and on internet, and emphasized in many scientific publications [7–12]. The U.S.Food and Drug Administration granted diacetyl GRAS (generally recognized as safe) status as a direct food ingredient, and consumption of the low levels of diacetyl present in food has not been reported to present a human health risk. However, the toxicity of inhaled diacetyl came under scrutiny after investigators from the National Institute for Occupational Safety and Health (NIOSH) reported in 2001 an outbreak of severe lung disease in popcorn-plant workers in Missouri [13–16]. In 2008, following recognition of potential risk to workers exposed to butter flavorings, 16 flavor companies requested consultation from investigators at the National Jewish Medical and Research Center to determine exposures and lung health risk among flavor manufacturing employees [17].

We have finally decided to broaden the scope of the study to 27 volatile compounds, commonly used by the industry and presenting potentially higher risks. In the present study, we developed a reliable and traceable method to evaluate occupational exposure risks to volatile chemical substances. We show that commercially available systems were not adapted or often underestimate the respiratory exposure compared to experimental data collected in the workplaces. We propose an improved model to evaluate more realistically occupational respiratory exposure and to implement more appropriate preventive measures. The new modeling system allows the establishment of individual and detailed exposure record card. We wish to propose the present method as an additional monitoring tool to improve respiratory health and safety in the flavor and fragrance manufacturing facilities.

## Materials and Methods

The study and all the procedure was approved by MANE's International Management Committee. The objectives and principles of the study were also presented and discussed with the Staff Representatives, members of the CHSCT (Comité d'Hygiène, de Sécurité et des Conditions de Travail—Committee for Health, Safety and Working Conditions) and of the Work Council (Comité d'Entreprise) as defined by the French Law. This work was also reported to the representatives of the French Working Authorities (Inspection du Travail).

Participants gave individually their verbal consent to participate the test, and the trapping devices were fastened on their clothes with their full agreement during the sampling period.

Written consent were not requested, as the study belongs to MANE's General Security Improvement Policy, established in agreement with the committees of staff representatives.

### Our approach

Studying the chemical risk in-house starts with listing of the chemicals used in the company and assessing their risk factor and, more precisely for this project, the inhalation risk.

In accordance with the regulations, the action taken by the employer through the general principles of chemical risk prevention, particularly involves removal of the hazardous product or process, substitution with a less hazardous product or procedure, reduction of the risk level when working on the procedure and the organization of staff training.

Collecting safety data sheets for each substance is the first action to be completed.

In these safety data sheets, the potential hazard is coded with an R risk or H hazard statement and an S safety phrase or P precautionary statement.

Starting with the R or H statements, the in-house regulatory department responsible for issuing safety data sheets for the mixture or preparations provides precautions for use for the worker through pictograms. These labeling rules are based on the Dangerous Substances Directive DSD [18], Dangerous Preparations Directive DPD [19] and on CLP Regulation 1272/2008 (European adaptation of the GHS) implemented in December 2010 [20].

Risk management starts with prevention, including traceability control, in order to determine the processes for the use of products, the workplaces where they are handled, and the people who handle them.

Data on the work stations in a workplace is also useful, as well as the inventory of available means of protection (general ventilation of the workplace, local exhaust ventilation, respiratory protective equipment).

On the basis of the above mentioned traceability, we initially carried out a risk prioritization taking into account:

- intrinsic hazards of the products used as identified in the safety data sheets provided by the suppliers
- frequency of use of the products
- analysis results carried out by specialized organizations (example: FEMA data [1]: Flavor & Extract Manufacturers Association)
- physical and chemical characteristics of products and the environment.

This first step led us to the drafting of a list of about forty substances to be monitored (S1 Table). Later, we added acetyl propionyl (2,3-pentanedione) to this list. It is occasionnaly used as a diacetyl substitute. We also included three volatile organic solvents (benzene, methanol and hexane) that are already subject to annual measurement via an accredited organization. These results from subcontractors were examined by our modeling system. It should be noted that benzene is only used at laboratory scale in order to quantify traces of this molecule, classified as a contaminant, in our raw materials.

To be able to respond adequately to the regulatory requirements in terms of personal exposure records, atmosphere samples were then taken in different workplaces in order to refine our risk knowledge.

These approaches led to a better exposure risk assessment for employees and made it possible to prioritize preventive actions which can be implemented in addition to those already in place.

### Institutional methods

Several standards refer to the methodology for quantification of volatile compounds present in workplace atmospheres. Some involve a stage of adsorption of molecules on a support followed by their recovery using an elution solvent [5, 21–22].

Others describe a similar phase of adsorption completed by thermal desorption [6, 23].

In all cases, a gas chromatograph equipped with a flame ionization (GC/FID) or mass spectrometry (GC/MS) detector is used to quantify signals and thereby the molecules.

Following reports related to exposure to diacetyl in the “popcorn” industry in the United States, two American organizations have published methods to quantify diacetyl in the workplace.

In the OSHA method [22], diacetyl is trapped on silica gel and then desorbed with a hydroalcoholic solution (Ethanol:95/Water:5). The entrapment is carried out in 2 tubes assembled in series with a sampling rate of 50 ml/min (total volume: 3 liters). The analysis is carried out by GC/FID and the assay is performed by internal calibration with p-cymene. This study highlighted the low impact of humidity level in the atmosphere on the recovery rate: for relative humidity close to 80%, the recovery rate is 94%.

In the NIOSH method [21] diacetyl is trapped on Anasorb^®^ CMS (Carbon Molecular Sieve) and is desorbed with an acetone—methanol (99:1) mixture. The sampling rate is between 10 and 200 ml/min (total volume: 10 liters). The analysis is carried out by GC/FID but, in this case, the assay is performed by external calibration. The latter method was completed in 2009 [24] by a study highlighting the considerable impact of humidity. For humidity close to 28%, the recovery rate of diacetyl varies between 70 and 96%. For humidity ranging from 28% to 50%, the recovery rate falls. It falls in the 10%–23% range if humidity exceeds 50%. These observations show that the results can be very much affected by ambient humidity depending on the nature of the selected support.

The influence of humidity is largely due to the nature of the absorbent and specifically to its hydrophilic character.

Consequently, this possible interference was the subject of very particular attention during the development of our analytical approach.

### Our methods

Our approach is outlined in 3 steps: adsorption of the molecules on a solid Tenax^®^ TA support, thermal desorption and analysis of the desorbate by GC/MS. Quantification is carried out by external calibration.

All samplings were performed using 3 tubes in series in order to guarantee the absence of breakthrough in the adsorption stage. The volume of breakthrough is specified by NF EN 1076 [6] standard as being the volume of atmosphere which can pass through the tubes, before the target quantity of substance recovered in the second sample tube exceeds 5% of the total recovered quantity. Consequently, if the target molecule quantity present in the second tube is higher than 5%, the sample must be invalidated.

We initially worked on a single molecule (diacetyl); then transposed the approach on a mixture containing an equal weight of diacetyl, acetyl propionyl and acetyl methyl carbinol.

The first tests on this mixture were carried out in the laboratory in order to determine the best conditions for sampling (nature of the adsorbent, pumping flow and duration, etc.) and of analysis.

Then, the conditions determined in the laboratory were tested in a dedicated place on our production site. This room has provided optimum, controlled conditions in terms of: “pollutant concentration/workroom” ratio, positioning of the sampling devices, volume of atmosphere sampled, absence of co-pollutants, pollutant/atmosphere exchange surface, etc…

Detailed results are given in S1 File. The method is repeatable, reproducible and linear.

In order to measure the effect of humidity on our results, MANE is equipped with an ATIS^™^ (Adsorb Tube Injector System, Sigma Aldrich), system consisting of a controlled humidity vaporization chamber. This equipment enabled us to vary the ratio of hygrometry during sampling in order to evaluate its impact.

The adsorbent that we selected as being the most effective (TENAX^®^ TA) is a distinctly hydrophobic adsorbent so theoretically scarcely influenced by the presence of humidity. However, the significant interference of hygrometry on the analytical results reported by the NIOSH [21, 24] led us to a more in-depth study of Tenax entrapment capabilities in a relative humidity ranging from 10 to 80% (S1 File).

Therefore, we were able to demonstrate experimentally that our method was very minimally influenced by significant changes in humidity in the temperature range 18–25°C.

Once developed and validated, the methods of sampling and analysis are being widespread in the various subsidiaries of the MANE group.

Considering that a significant time period will elapse between the sampling performed in the subsidiaries and the tests performed in Le Bar-sur-Loup site, we have also carried out tests to estimate the maximum shelf life of our sample tubes before analysis. These tests revealed a good conservation of our samples over a period of up to 16 days before analysis. Moreover we did not detect significant degradation of our three target molecules over this period (S1 File).

A tightness test was successfully performed in operating conditions much more severe than those recommended by NF EN 1076 standard [6]. Despite these extreme conditions, the devices were found to be impermeable in compliance with the standard specifications, thereby ensuring the sample integrity and absence of cross-contamination when transporting these tubes.

Taking into account our industrial environment, the approach was extended to other molecules presenting a respiratory risk and used in high volumes.

A procedure for performing sampling was formalized. Five methods were developed depending on the molecules to be quantified. A summary of these methods is given in S2 Table.

## Commercially Available Modeling Softwares

An examination of the literature shows that, to date, several models were developed to evaluate consumer, environmental or operator exposure. The main models are:

- CONSEXPO: mathematical model for evaluation of exposure to compounds present in consumer products. The model was developed by the RIVM (National Institute of the Public Health and the Environment in the Netherlands) in order to be able to estimate and evaluate exposure to substances from consumer products following their absorption by the respiratory, oral or dermal route [25].
- TWO-BOX MODEL: tool developed by the RIFM (Research Institute for Fragrance Materials) to predict simply, so far not taking account the physical and chemical specificities of the various substances, consumer exposure related to “air fresheners”, candles or aerosols, by considering two levels of exposure materialized by two boxes. This tool is the subject of a study to improve its performance while retaining its simplicity [26–27].
- ECETOC-TRA (European Centre for Ecotoxicology and Toxicology Of Chemicals—Target Risk Assessment): a tool which provides a staged approach to calculating the exposure and the risks of a chemical substance, and covers consumer, environmental or employee exposure. It was developed by ECETOC [28].
- EUSES (European Union System for the Evaluation of Substances): a decision-making tool which allows fast, effective assessments of risks generated by chemicals. EUSES makes it possible to balance the “intrinsic danger of a substance versus exposure”, However, it requires a lot of input data [29].
- STOFFENMANAGER (or “substance manager” in Dutch): originally an Internet tool created for small and medium-size companies, in order to define the priorities of the various identified risks [30].

We have examined the ability of these models for evaluating operator exposure in our industry.

They were mostly developed to assess the exposure of consumers to finished products (lacquers, air-fresheners, etc.) in particular as part of the application of the REACH regulation. Some models require understanding of a very large amount of input data (example: EUSES) and without these values, the use of the model default values leads to a very inaccurate, incomplete exposure assessment.

We were looking for a model that was easy to use, robust, reliable and suitable for the many substances used in the flavour and fragrance industry. We selected the ECETOC-TRA (version 3) model for its operating principle and its adaptability to our business.

## Results

### General parameters involved in modeling system

MANE was guided in its modeling approach by 2 important goals:

1. To be as close as possible to real working conditions, by taking into account specific parameters of the working environment:
  - exposure frequency (number of operations over a given period of time)
  - evaluation of the concentration of the chemical in the respiratory surrounding of the operator
  - taking into account the workplace environment and the collective and individual preventive measures in place.
2. To reflect global working characteristics of the Fragrance and Flavour industry:
  - a very large number of chemical compounds used by a worker during a given period (on average a worker performs 12,500 weighing operations of chemicals in a year)
  - generally a very short exposure time because of the small quantities involved in the formulation.

The model proposed by MANE can provide an operator exposure assessment during the use of a chemical identified as hazardous by inhalation or a preparation containing this kind of substance. It is based on the operating principle of the ECETOC-TRA model in which the modelled exposure is a function of the vapor pressure of the substance and is modulated by correction coefficients. Our methodology was, in a first step, to optimize the correlation between vapor pressure and initial exposure; and in a second step to apply correction coefficients, to more accurately take into consideration the real parameters of the different working places, as detailed hereafter.

The inhalation hazard characteristics for health follow the DSD and/or CLP classification rules. It may be considered that a chemical agent presents a health risk by inhalation once its classification contains at least one of the following risks phrases (S3 Table).

The model proposed here does not apply to exposure by skin contact or ingestion. We can consider an absence of exposure to the skin or oral route in the production workplace once the use of the substances concerned takes place while wearing personal protective equipment that is compulsory for company staff: gloves, protective clothing, safety glasses, protection masks.

**Fig 1:**
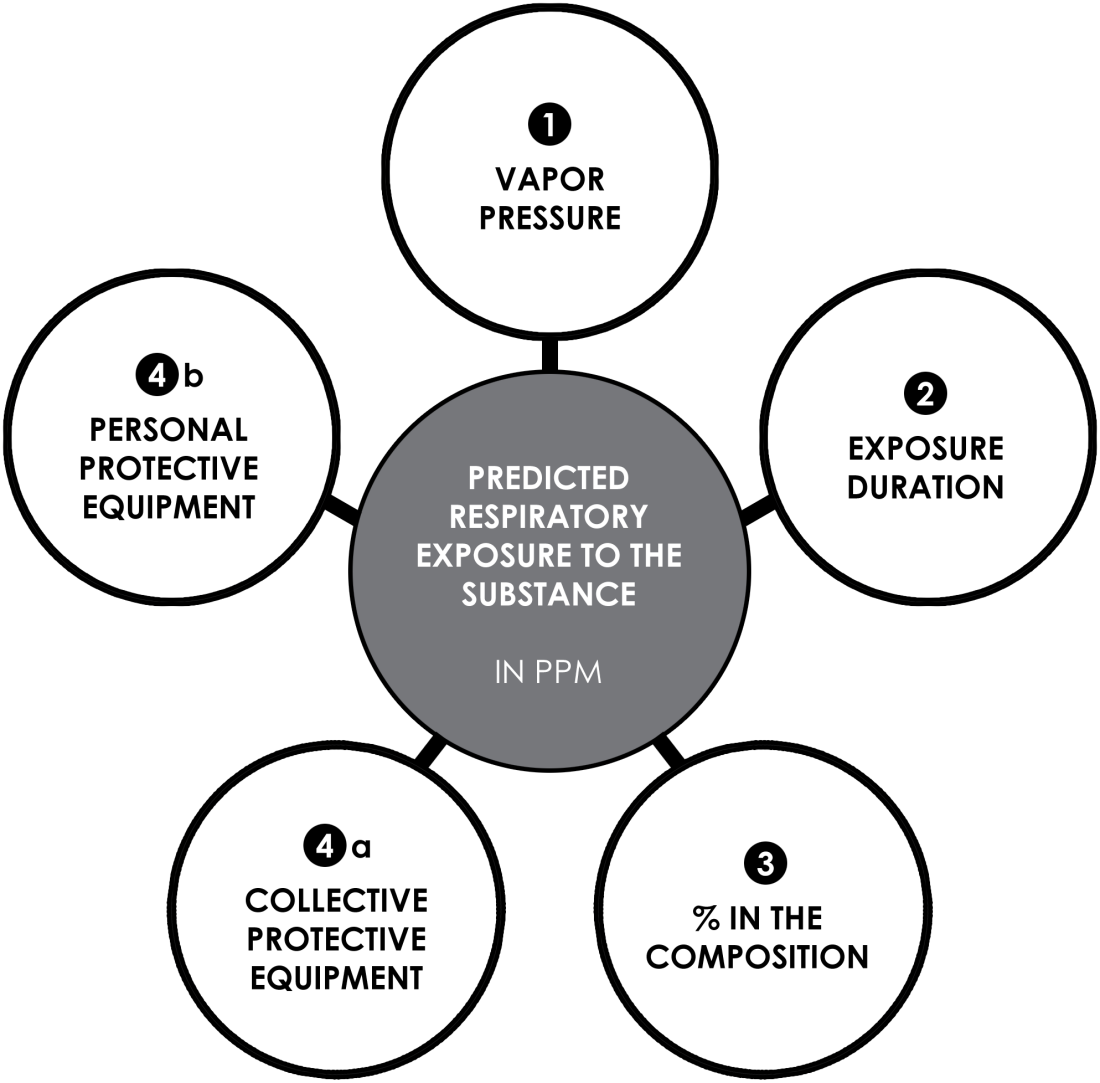
Parameters involved in models.

The model is based on input data used by almost all the models examined (ECETOC TRA, EUSES, CONSEXPO), namely (Fig 1):

1. the vapor pressure of the substance used: the higher it is, the higher the emission rate of the vapors, and the higher the initial exposure will be
2. the exposure duration: this is the time during which the substance is used outside of its container
3. the percentage of the substance in the composition (100% for pure substances)
4. the operating conditions used by the operator;
  - the presence or absence of collective protective equipment: local exhaust ventilation
  - the wearing or not of personal protective equipment: filtering respiratory protection mask

The modelled exposure is calculated starting from an initial exposure to which reduction coefficients (correction factors), for each criterion, are applied:

- exposure duration: coefficient A
- % of the substance in the composition: coefficient B
- presence of collective protective equipment: coefficient C
- use of personal protective equipment: coefficient D

Modelled Exposure=Initial Exposure × coeff.A × coeff.B × coeff.C × coeff.D

**Fig 2:**
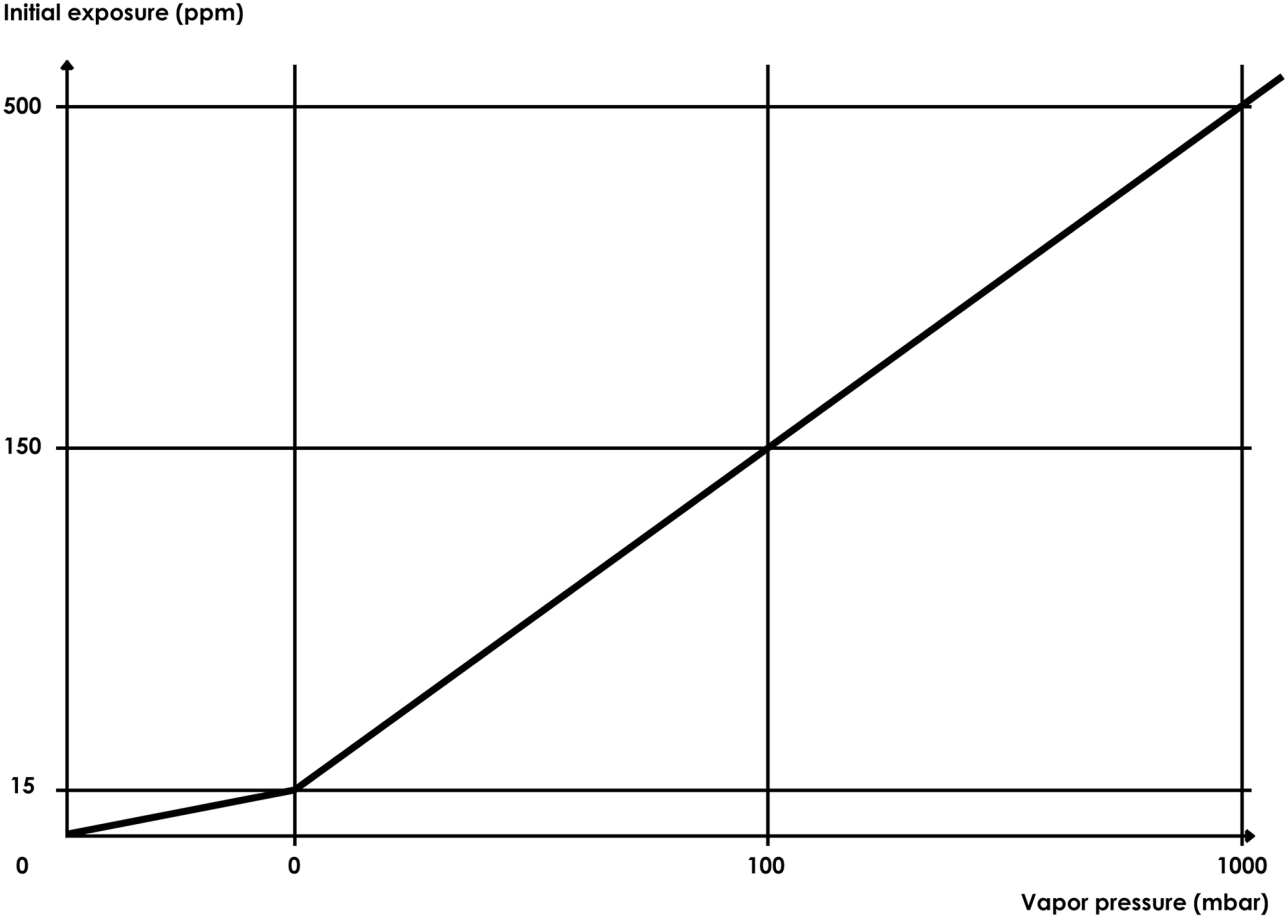
Curve initial exposition vs vapor pressure.

The determination of the initial exposure is a function of the vapor pressure of the substance at room temperature expressed in millibars (mbars) according to the following curve (Fig 2):

This curve was drawn using the VP-initial exposure relationship given by the ECETOC TRA model adjusted to the results of the measurements carried out in real situation.

**Fig 3:**
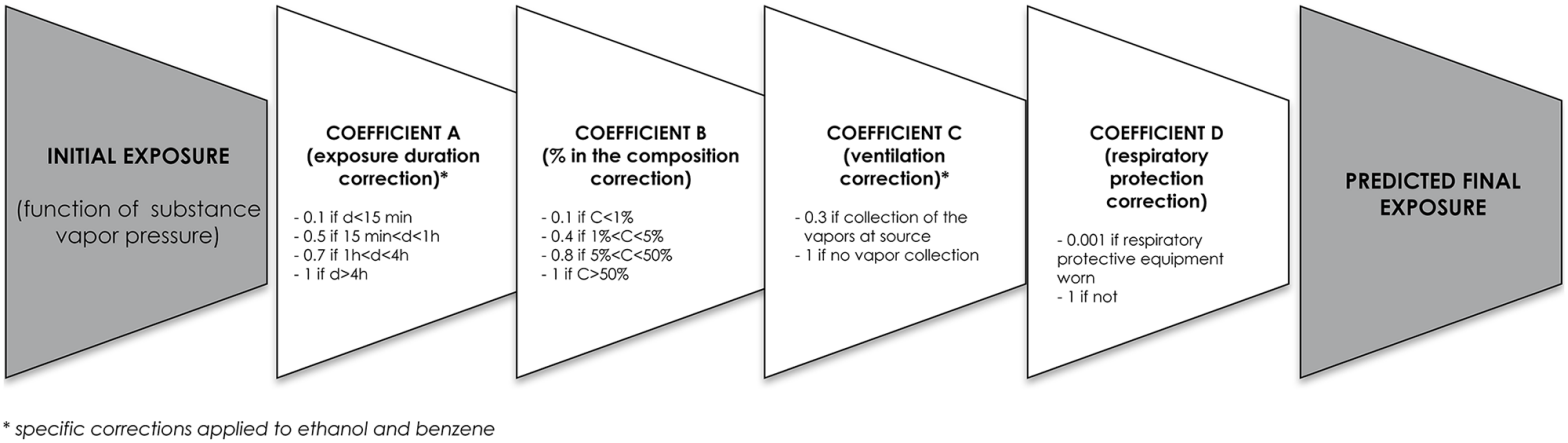
Correction parameters for model development.

The determination of the various coefficients A, B, C, D is based on the data proposed by ECETOC-TRA and modified according to our experimental results (Fig 3):

### Specific cases

**Fig 4:**
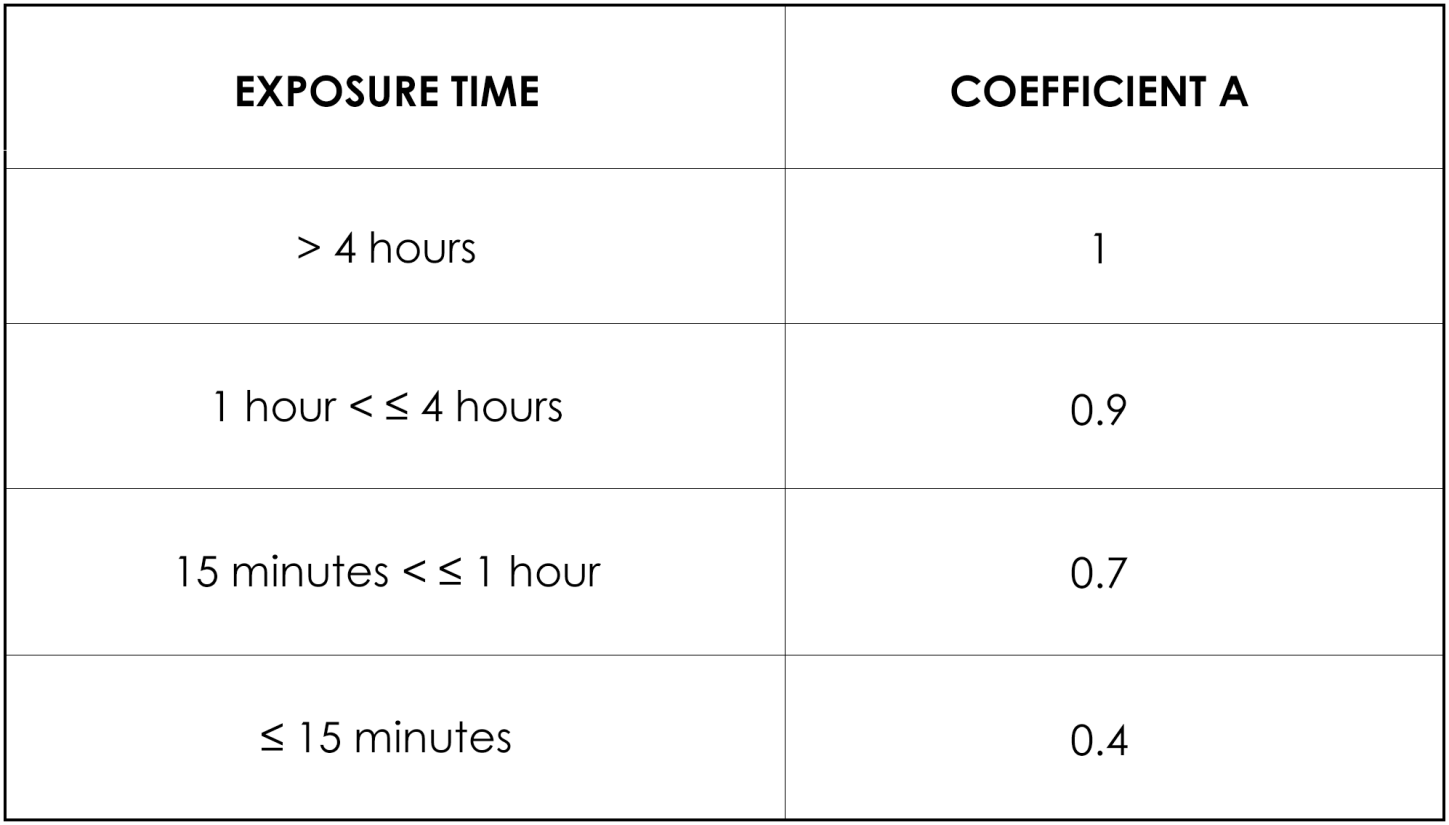
Correction factors of exposure times in the special case of ethanol.

Ethanol (CAS N° 64-17-5) is a substance widely used in the Fragrance and Flavor industry, in particular in the manufacture of compositions intended for the food industry. Measurements of exposure in real situations performed in various scenarios of ethanol use showed that the model previously described underestimated the real exposure. A model specific to ethanol was thus produced with specific values: initial exposure set at 200 ppm and variation of coefficient A for exposure duation according to the values given in Fig 4.

Benzene (CAS N° 71.43.2) is a forbidden substance, which is only manipulated in laboratory conditions (under appropriate hoods), and never in production work places. As a consequence, our modeling system over-evaluated the exposure to benzene. Based on our experimental results and model parameters, an adjusted value for Coefficient C was needed for benzene. The chosen value is 0.03.

### Evaluation of our model

The verification of the validity of the model is based on the comparison of the values modelled in various exposure scenarios with the experimental values measured in real situations.

As this data is representative of operator exposure level to chemical agents likely to have an impact on health, it is important that the model put in place does not underestimate the exposure level.

In order to check the validity of its model, we monitored more than 430 solvents measurements of exposure in different workplaces to represent as closely as possible the working environnement (S4 Table):

- during the use of 27 substances: the most used among the forty preselected substances
- during various operations involving these substances. weighing and mixing (54.5%, both operations are always associated in production facilities), packaging (32.5%) and reconditioning/transferring (11.6%).

The examination of the table of the results shows that, for a given substance, in spite of a priori identical working conditions, experimental results can unexpectedly vary. This observation might be explained by the multitude and unpredictability of parameters related to handling behavior (lack of experience of the operator, etc.), accidental events (mishandling, etc.) and environment variations (positioning towards air vent, air movement due to human traffic, etc.) which cannot be integrated in an exhaustive way into a modelized approach.

After examination of the results (S4 Table), 98% of the values obtained with our model are above the experimental values measured in real conditions. On the other hand, only 37% of the values obtained with the ECETOC-TRA method are above experimental values. Considering the high variability of the working conditions, as mentioned above, the overestimation generated by our model gives a wider safety margin, and corroborates our willingness not to underestimate the occupational exposure. Our goal was to select assumptions about exposure that do not lead to underestimation of exposure. We must not forget that the purpose of the current study was to improve safety of workers in the flavor and fragrance industry, and we believe our model achieves the desired purpose, even at the cost of an overevaluation of real exposure.

## Discussion

The particular characteristic of the Fragrance and Flavor industry concerning chemical risk assessment lies in the repeated use of a large number of different chemicals in order to produce compositions by blending. Approximately 50% of the chemicals used are hazardous chemicals: they present at least one health risk (by inhalation, contact or ingestion) and must thus be listed in the individual exposure record card. Approximately 25% of the hazardous chemicals used are hazardous by inhalation.

Given the specific nature of the Fragrance and Flavour industry, characterized by the daily use of many chemicals in many scenarios and with possible different exposures to the same agent, it does not seem possible to indicate routinely in the personal exposure record card, for each employee, the exposure level established from measurements performed in real conditions. Thus the interest of modeling is obvious and unquestionable.

From the data:

1. Regulatory: hazard symbols and risk statements
2. Physical and chemical: vapor pressure
3. For production over a given period of time: name of the operator, dates from the operation, name and quantity of the chemical used, identification of the work station used
4. Relating to the work station or the operating conditions: local exhaust ventilation and use of personal protective equipment.

Our proposed model allows the generation of annual personal exposure record card including the name of the operator, the type of operation carried out, the name of the chemicals, the collective and individual preventive measures used, the unit operation duration, the number of operations carried out in the given period of time, the substance content in the composition, and finally the exposure assessment for the operation.

For the experimental section, we must keep in mind that the limits of this analytical technique lie in the significant number of factors that may interfere both during sampling and analysis:

- Presence of unforeseeable co-pollutants (molecules not studied but present in large quantity in the atmosphere): there could be a phenomenon of competition at the adsorption step and induction of suppressions and variations in split flows during the thermal desorption analysis affecting the repeatability and consequently the accuracy of the analyses. The places where sampling is done differ greatly and the real conditions cannot be anticipated. If the conditions met in another plant are very different from those encountered when developing the method, it will be necessary to make sure that the method remains applicable.
- Room volume/concentration of the examined solution ratio: depending on the atmospheric concentration of the target molecules and volume of the room, the sampling volume could prove to be too large (saturation of the sampling devices) or on the contrary too low to reach the threshold of detection and/or quantification. It is not possible to anticipate real parameters. Only a linearity study will show a saturation phenomenon.
- If the temperature is very different from that encountered when the method was developed (18 to 25°C) the saturation of the tubes will be accelerated, the time and/or the pumping flow may be too great, thus accelerating the possible saturation of the sampling devices.

The findings, within the framework of this work, agree with the limitations of this analytical approach described in the standard, AFNOR X43-267 [5]. To mitigate this interference this same standard recommends routinely validating the analytical approaches and methodologies of sampling, both depending on new molecules (and/or mixtures) studied and also to take into account the real parameters met in production: an approach which we adhered to for our entire analytical protocol.

The analysis of the results (S4 Table) shows that the predictions of exposure obtained by our proposed model better correlate with the measured values. This correlation is particularly well improved in the case of the use of respiratory protection masks (Coefficient D = 0.001). It highlights and confirms the importance of wearing masks for operators when handling substances presenting a chronic health risk.

## Conclusion

The modeling method developed here is a tool of prevention which supports decision-making processes in order to reduce opportunities for hazardous exposures, to implement adequate personal and/or collective protective equipments, and to train employees in a more targeted way. As recommended by the FEMA, it could help the design of exposure controlling measures in the fields of facility structure, working organization, material storage, personal respiratory protection, ventilation, packaging choices, automatization of processes, etc. [1].

The present measurement campaign has already driven some practical and positive actions in the workshops at MANE. One might give the example of the promotion of smaller containers in order to minimize volatilization during transfer operations, but also the handling of the most dangerous substances at the end of the weighing and mixing processes. These are small, but significant steps towards safer and healthier workplaces in the flavor and fragrance industry.

The initial implementation of this model is the “foundation” of a large-scale business project for continuous improvement over the coming years:

- it is being applied gradually to the other french sites and the subsidiaries of the MANE Group
- it takes into account of the continuous optimization of the sampling and analysis methods
- it is a source of information for the implementation of actions to improve employees’ health: respiratory function examinations, upgrade of the chemical risk training, design of modifications and improvements of the work stations
- it must be adapted to all changes: legislative, regulatory, technical or organizational.

Very recently, different authors worked on volatile organic compounds released during roasting and grinding of coffee [31–32]. They published exposure values for diacetyl and acetyl propionyl that may exceed recommended exposure professional limits proposed by NIOSH and the European Commission [11]. These results may be useful in understanding the potential risks of respiratory disease from various volatile organic compounds and illustrate that it is not an easy task to recommend exposure limit values.

## Supporting Information

S1 File Validation of the sampling method for diacetyl, acetyl propionyl and acetyl methyl carbinol. Includes data on repeatability, linearity, the influence of hygrometry and impermeability of sampling devices. Analyzes parameters impacting sampling operations and storage of the sampling tubes.
(DOC)

S1 Table List of volatile organic substances selected and analyzed in the present study. Chemical names and CAS numbers of the 27 substances selected for the reason that they are handled in large amounts and represent potentially higher risks.
(DOCX)

S2 Table MANE sampling and analytical methods. Full detail of the nature of the absorbents, the pumping and desorption parameters, and analytical methodologies.
(DOCX)

S3 Table Risk phrases. The volatile compounds that were quantified in the present study were selected for their potentially high inhalation and respiratory risks, according to the risks phrases listed here. R-phrases and their corresponding symbols according to DSD: Dangerous Substances Directive (Directive 67/548/CEE); DPD: Dangerous Preparations Directive (Directive 1999/45/CE) and CLP Regulation (EC Regulation 1272/2008) are presented here in a table form.
(DOCX)

S4 Table Full data table. Comprehensive sample results collected in V. MANE FILS workshops.
(DOCX)
