# Supplementary material for: Respiratory Health – Exposure Measurements and Modeling in the Fragrance and Flavour Industry
Source: PLoS One. 2016 Feb 10;11(2):e0148769. doi: 10.1371/journal.pone.0148769 (PMC4749324; doi:10.1371/journal.pone.0148769)
Supplement: S1 File — Includes data on repeatability, linearity, the influence of hygrometry and impermeability of sampling devices. Analyzes parameters impacting sampling operations and storage of the sampling tubes. (DOC) [file pone.0148769.s001.doc]

**S1 File. VALIDATION OF THE SAMPLING METHOD FOR DIACETYL, ACETYL PROPIONYL AND ACETYL METHYL CARBINOL.**

**Contents :**

1. ***REPEATABILITY***
   1. **"Methanol" Matrix**
   2. **"Ethanol" Matrix**
2. ***LINEARITY***
   1. **"Methanol" Matrix**
   2. **"Ethanol" Matrix**
3. ***THE INFLUENCE OF HYGROMETRY***
   1. **Calibration of ATIS^TM^ System**
   2. **IMPACT OF HYGROMETRY ON SAMPLING OPERATIONS**
      1. **Impact of hygrometry on a 5 minutes sampling time.**
      2. **Impact of hygrometry on a 20 minutes sampling time.**
      3. **Impact of hygrometry on a 70 minutes sampling time.**
4. ***IMPERMEABILITY OF SAMPLING DEVICES***
5. ***BREAKTHROUGH OF SAMPLING DEVICES WITH A FIXED FLOW RATE ANALYSIS (50 ml/min)***
6. ***STORAGE STUDY***
7. ***SAMPLING TESTS IN A CLOSED CHAMBER***
8. ***SAMPLING TESTS IN A DEDICATED PLACE ON OUR PRODUCTION PLANT***
   1. **Study of the influence of the variation pumping time on the quantity of pollutants sampled.**
   2. **Impact of the studied solution concentration.**
9. ***REPEATABILITY.***

For the establishment of our analytical model, and more especially the choice of dilution solvent for our standards, the tables referencing breakthrough volumes by solvents and the type of adsorbents recommended that we use a methanol matrix rather than ethanol for their preparation. The use of methanol as a dilution medium for our standards would seem to be a wiser choice with regard to its much lower breakthrough volume than that of ethanol, thus allowing it to be almost totally eliminated during pumping. In fact, its elimination from the traps as a result of its low breakthrough volume makes it possible firstly to avoid the saturation of the tube with methanol and thus disruption of the thermal desorption of our analyses by creating segregation in the trap due to over-pressure as it moves to the vapor phase, and secondly avoids causing variations in the split flow rate likely to cause repeatability problems.

Moreover, we observed and confirmed, during our tests, that ethanol, due to its physical properties and its volatility close to that of our compounds, created disturbances.

However, as ethanol is a widely used molecule in our industry and our workplaces, it seemed necessary to develop a method in order to minimize as far as possible the interferences generated by its presence. To check this non-disturbance of the “ ethanol ” matrix, we conducted a double study and compared the results obtained on the two matrices.

**I-1 Standard Solution: 100 ng/µl prepared in Methanol.**

Study carried out with GILIAN 5000 and 3500 pumps on 10 successive injections of 1µl of a standard solution prepared so that a deposit of 1µl of this corresponds to 100 ng of each of our three molecules (Diacetyl/Acetyl Propionyl/Acetyl Methyl Carbinol) deposited on the tube.

- Sampling time: 5 minutes
- Propellent gas flow rate: 50 ml/min

Pump


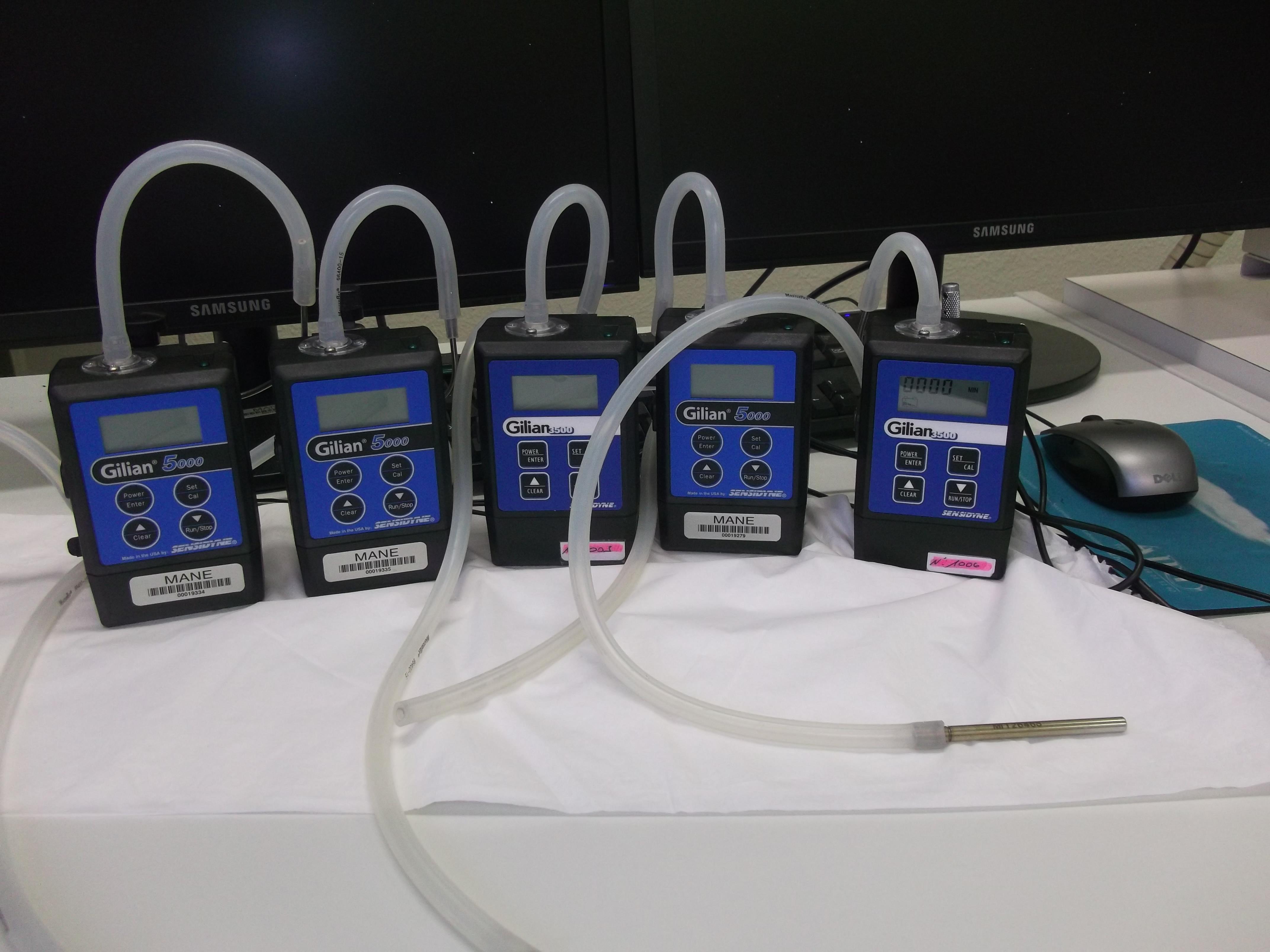


|  |  | | **TUBE 1** | **TUBE 2** | **TUBE 3** | **TUBE 4** | **TUBE 5** | **TUBE 6** | **TUBE 7** | **TUBE 8** | **TUBE 9** | **TUBE 10** | **AVERAGE** | **STD**  **DEVIATION** | **CV %** |
| --- | --- | --- | --- | --- | --- | --- | --- | --- | --- | --- | --- | --- | --- | --- | --- |
| **DIACETYL** | | 3790357 | | 3791014 | 3961897 | 3851946 | 3811902 | 3660164 | 3844103 | 3888221 | 3738222 | 3858033 | 3819585,9 | 82902,1 | 2,17 |
| **ACETYL PROPIONYL** | | 945915 | | 952157 | 958091 | 959945 | 944923 | 938658 | 953883 | 948247 | 925285 | 943246 | 947035 | 10147,9 | 1,07 |
| **ACETYL METHYL CARBINOL** | | 1309935 | | 1323050 | 1238698 | 1318606 | 1279499 | 1335130 | 1344858 | 1309739 | 1284675 | 1311851 | 1305604,1 | 30834,4 | 2,36 |

**I-2 Standard Solution: 100 ng/µl prepared in Ethanol.**

|  | **TUBE 1** | **TUBE 2** | **TUBE 3** | **TUBE 4** | **TUBE 5** | **TUBE 6** | **TUBE 7** | **TUBE 8** | **TUBE 9** | **TUBE 10** | **AVERAGE** | **STD**  **DEVIATION** | **CV %** |
| --- | --- | --- | --- | --- | --- | --- | --- | --- | --- | --- | --- | --- | --- |
| **DIACETYL** | 6186313 | 6070150 | 6130375 | 5756120 | 6068195 | 6262572 | 6476177 | 5902294 | 6051847 | 6005825 | 6090986,8 | 196052,8 | 3,22 |
| **ACETYL PROPIONYL** | 1498789 | 1487254 | 1476951 | 1505094 | 1481869 | 1479302 | 1464808 | 1430080 | 1453250 | 1462117 | 1473951,4 | 22213,5 | 1,51 |
| **ACETYL METHYL CARBINOL** | 1730084 | 1583513 | 1667746 | 1981732 | 1667915 | 1531946 | 1344674 | 1531239 | 1522046 | 1687581 | 1624847,6 | 168073,7 | 10,34 |

Conclusion: We therefore confirmed by our tests that methanol is more easily eliminated from the trap, due to its low breakthrough volume, and thus decreases the disturbances generated by a saturation phenomenon in the sampling mediums.

1. ***LINEARITY***

**II-1 Standard Solution: 100 ng/µl prepared in Methanol.**

Preparation of a wide range of standard Solutions of Diacetyl, Acetyl propionyl and Acetyl methyl carbinol at approximately exactly 0.1, 1, 10, 50, 100, 200 and 400 ng in methanol. Five samples of each standard solution were taken using GILIAN pumps for 5 minutes at 50 ml/min.

Results:

| **0,1 ng** | | **TUBE 1** | **TUBE 2** | **TUBE 3** | **TUBE 4** | **TUBE 5** | **AVERAGE** | **STD DEVIATION** | **CV %** |
| --- | --- | --- | --- | --- | --- | --- | --- | --- | --- |
| **DIACETYL** | | 45437 | 54416 | 68107 | 41578 | 100212 | 61950 | 23697,8 | 38,25 |
| **ACETYL PROPIONYL** | | 2640 | 3546 | 5134 | 2676 | 6550 | 4109,2 | 1698,3 | 41,33 |
| **ACETYL METHYL CARBINOL** | | 12638 | 7629 | 7712 | 4952 | 15521 | 9690,4 | 4280,8 | 44,18 |
|  |  |  |  |  |  |  |  |  |  |
|  |  |  |  |  |  |  |  |  |  |
| **1 ng** | | **TUBE 1** | **TUBE 2** | **TUBE 3** | **TUBE 4** | **TUBE 5** | **AVERAGE** | **STD DEVIATION** | **CV %** |
| **DIACETYL** | | 64885 | 70536 | 71088 | 75506 | 73507 | 71104,4 | 4004,7 | 5,63 |
| **ACETYL PROPIONYL** | | 11683 | 10172 | 10210 | 10393 | 10481 | 10587,8 | 625,4 | 5,91 |
| **ACETYL METHYL CARBINOL** | | 15087 | 10355 | 9678 | 9580 | 11051 | 11150,2 | 2278,9 | 20,44 |
|  |  |  |  |  |  |  |  |  |  |
|  |  |  |  |  |  |  |  |  |  |
| **10 ng** | | **TUBE 1** | **TUBE 2** | **TUBE 3** | **TUBE 4** | **TUBE 5** | **AVERAGE** | **STD DEVIATION** | **CV %** |
| **DIACETYL** | | 545817 | 466618 | 465992 | 438637 | 462500 | 475912,8 | 40745,6 | 8,56 |
| **ACETYL PROPIONYL** | | 93045 | 94288 | 94333 | 92412 | 98184 | 94452,4 | 2242,2 | 2,37 |
| **ACETYL METHYL CARBINOL** | | 76835 | 144245 | 122374 | 133342 | 139205 | 123200,2 | 27167,6 | 22,05 |
|  |  |  |  |  |  |  |  |  |  |
|  |  |  |  |  |  |  |  |  |  |
| **50 ng** | | **TUBE 1** | **TUBE 2** | **TUBE 3** | **TUBE 4** | **TUBE 5** | **AVERAGE** | **STD DEVIATION** | **CV %** |
| **DIACETYL** | | 1690790 | 1614881 | 1739025 | 1714643 | 1778102 | 1707488 | 61006,1 | 3,57 |
| **ACETYL PROPIONYL** | | 413156 | 395592 | 416020 | 415091 | 414850 | 410941,8 | 8642,9 | 2,10 |
| **ACETYL METHYL CARBINOL** | | 624781 | 609824 | 625945 | 622410 | 586520 | 613896 | 16599,5 | 2,70 |
|  |  |  |  |  |  |  |  |  |  |
|  |  |  |  |  |  |  |  |  |  |
| **100 ng** | | **TUBE 1** | **TUBE 2** | **TUBE 3** | **TUBE 4** | **TUBE 5** | **AVERAGE** | **STD DEVIATION** | **CV %** |
| **DIACETYL** | | 3790357 | 3791014 | 3961897 | 3851946 | 3811902 | 3841423 | 71841,9 | 1,87 |
| **ACETYL PROPIONYL** | | 945915 | 952157 | 958091 | 959945 | 944923 | 952206,2 | 6840,1 | 0,72 |
| **ACETYL METHYL CARBINOL** | | 1309935 | 1323050 | 1238698 | 1318606 | 1279499 | 1293958 | 35255,7 | 2,72 |
|  |  |  |  |  |  |  |  |  |  |
|  |  |  |  |  |  |  |  |  |  |
| **200 ng** | | **TUBE 1** | **TUBE 2** | **TUBE 3** | **TUBE 4** | **TUBE 5** | **AVERAGE** | **STD DEVIATION** | **CV %** |
| **DIACETYL** | | 7313866 | 7677587 | 7521822 | 7863519 | 7680697 | 7611498 | 205728,8 | 2,70 |
| **ACETYL PROPIONYL** | | 1919981 | 1946841 | 1893335 | 1890280 | 1924685 | 1915024 | 23520,8 | 1,23 |
| **ACETYL METHYL CARBINOL** | | 2834749 | 2771928 | 2640842 | 2412875 | 2725002 | 2677079 | 163760,2 | 6,12 |
|  |  |  |  |  |  |  |  |  |  |
|  |  |  |  |  |  |  |  |  |  |
| **400 ng** | | **TUBE 1** | **TUBE 2** | **TUBE 3** | **TUBE 4** | **TUBE 5** | **AVERAGE** | **STD DEVIATION** | **CV %** |
| **DIACETYL** | | 1,4E+07 | 1,5E+07 | 1,5E+07 | 1,5E+07 | 1,5E+07 | 14743833 | 309995,0 | 2,10 |
| **ACETYL PROPIONYL** | | 3903241 | 4016939 | 4006251 | 4014451 | 3975406 | 3983258 | 47698,2 | 1,20 |
| **ACETYL METHYL CARBINOL** | | 5819119 | 6127643 | 6004939 | 6059159 | 5962915 | 5994755 | 115944,6 | 1,93 |

| **DIACETYL** | | | | | | |
| --- | --- | --- | --- | --- | --- | --- |
| **CONTENT** | 0,99 | 9,9 | 49,5 | 99,00 | 198,00 | 396,00 |
| **AREA** | 95621,2 | 563091,00 | 2012261,00 | 3850375,6 | 8284084,4 | 14790552,4 |


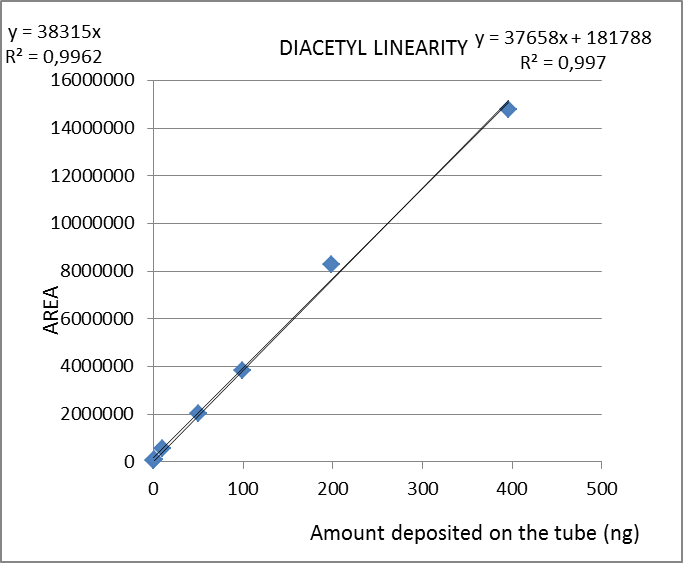


| **ACETYL PROPIONYL** | | | | | | |
| --- | --- | --- | --- | --- | --- | --- |
| **CONTENT** | 0,957 | 9,57 | 47,85 | 95,7 | 191,4 | 382,8 |
| **AREA** | 14411,4 | 141510,6 | 497116 | 1031330,2 | 2244280,6 | 4069489 |

**
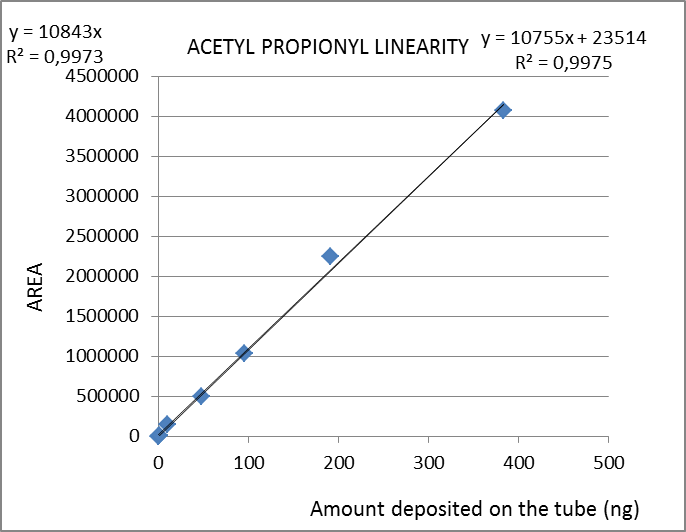
**

| **ACETYL METHYL CARBINOL** | | | | | | |
| --- | --- | --- | --- | --- | --- | --- |
| **CONTENT** | 1 013 | 10,13 | 50,65 | 101,3 | 202,6 | 405,2 |
| **AREA** | 18164,4 | 191537 | 731941 | 1538613,8 | 3416038 | 6376038,8 |


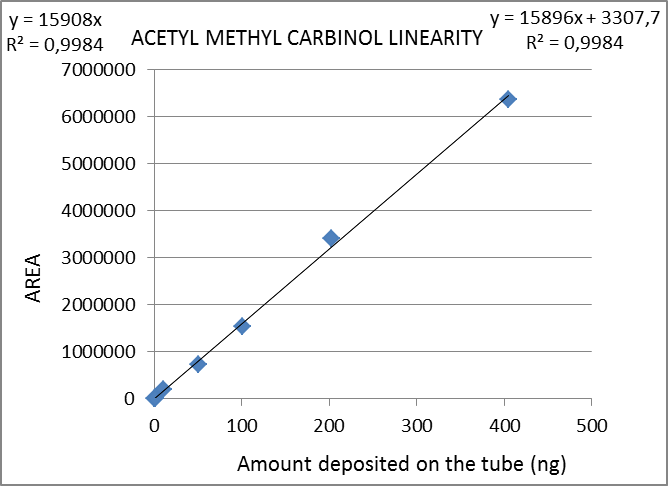


**II-2 Standard Solution: 100 ng/µl prepared in Ethanol.**

Preparation of a wide range of standard Solutions of Diacetyl, Acetyl propionyl and Acetyl methyl carbinol at approximately 0.1, 1, 10, 50, 100, 200 and 400 ng in methanol. Five samples of each standard solution were taken using GILIAN pumps for 5 minutes at 50 ml/min.

Results:

| **0,1 ng** | | **TUBE 1** | **TUBE 2** | **TUBE 3** | **TUBE 4** | **TUBE 5** | **AVERAGE** | **STD DEVIATION** | **CV %** |
| --- | --- | --- | --- | --- | --- | --- | --- | --- | --- |
| **DIACETYL** | | 187647 | 63105 | 64403 | 164153 | 95281 | 114917,8 | 57739,8 | 50,24 |
| **ACETYL PROPIONYL** | | 12877 | 4991 | 4566 | 10646 | 5088 | 7633,6 | 3854,9 | 50,50 |
| **ACETYL METHYL CARBINOL** | | 5201 | 6443 | 5465 | 7573 | 5153 | 5967 | 1037,7 | 17,39 |
|  |  |  |  |  |  |  |  |  |  |
| **1 ng** | | **TUBE 1** | **TUBE 2** | **TUBE 3** | **TUBE 4** | **TUBE 5** | **AVERAGE** | **STD DEVIATION** | **CV %** |
| **DIACETYL** | | 96515 | 91173 | 91141 | 113742 | 107834 | 100081 | 10231,4 | 10,22 |
| **ACETYL PROPIONYL** | | 13759 | 13772 | 13172 | 14539 | 13260 | 13700,4 | 544,3 | 3,97 |
| **ACETYL METHYL CARBINOL** | | 17459 | 18450 | 15934 | 19674 | 17974 | 17898,2 | 1370,6 | 7,66 |
|  |  |  |  |  |  |  |  |  |  |
| **10 ng** | | **TUBE 1** | **TUBE 2** | **TUBE 3** | **TUBE 4** | **TUBE 5** | **AVERAGE** | **STD DEVIATION** | **CV %** |
| **DIACETYL** | | 561367 | 513958 | 521813 | 694483 | 577767 | 573877,6 | 72488,6 | 12,63 |
| **ACETYL PROPIONYL** | | 113990 | 118135 | 118232 | 124609 | 122272 | 119447,6 | 4111,1 | 3,44 |
| **ACETYL METHYL CARBINOL** | | 96596 | 173399 | 149009 | 121570 | 164380 | 140990,8 | 31661,1 | 22,46 |
|  |  |  |  |  |  |  |  |  |  |
| **50 ng** | | **TUBE 1** | **TUBE 2** | **TUBE 3** | **TUBE 4** | **TUBE 5** | **AVERAGE** | **STD DEVIATION** | **CV %** |
| **DIACETYL** | | 1852837 | 1891305 | 1878553 | 1912716 | 2063259 | 1919734 | 83108,3 | 4,33 |
| **ACETYL PROPIONYL** | | 456983 | 448902 | 445887 | 462373 | 455462 | 453921,4 | 6575,0 | 1,45 |
| **ACETYL METHYL CARBINOL** | | 704189 | 662195 | 670532 | 690808 | 592062 | 663957,2 | 43459,2 | 6,55 |
|  |  |  |  |  |  |  |  |  |  |
| **100 ng** | | **TUBE 1** | **TUBE 2** | **TUBE 3** | **TUBE 4** | **TUBE 5** | **AVERAGE** | **STD DEVIATION** | **CV %** |
| **DIACETYL** | | 2877873 | 2947515 | 2675296 | 2871957 | 2982196 | 2870967,4 | 118919,4 | 4,14 |
| **ACETYL PROPIONYL** | | 698566 | 709403 | 687670 | 702858 | 698434 | 699386,2 | 7925,3 | 1,13 |
| **ACETYL METHYL CARBINOL** | | 1233741 | 1241987 | 1180945 | 1229314 | 1178307 | 1212858,8 | 30690,4 | 2,53 |
|  |  |  |  |  |  |  |  |  |  |
| **200 ng** | | **TUBE 1** | **TUBE 2** | **TUBE 3** | **TUBE 4** | **TUBE 5** | **AVERAGE** | **STD DEVIATION** | **CV %** |
| **DIACETYL** | | 5448148 | 5498405 | 5499431 |  | 5439470 | 5471363,5 | 32016,6 | 0,59 |
| **ACETYL PROPIONYL** | | 1277677 | 1283221 | 1283080 |  | 1290396 | 1283593,5 | 5218,0 | 0,41 |
| **ACETYL METHYL CARBINOL** | | 2486224 | 2569045 | 2508685 |  | 2615291 | 2544811,25 | 58572,6 | 2,30 |
|  |  |  |  |  |  |  |  |  |  |
| **400 ng** | | **TUBE 1** | **TUBE 2** | **TUBE 3** | **TUBE 4** | **TUBE 5** | **AVERAGE** | **STD DEVIATION** | **CV %** |
| **DIACETYL** | | 9211856 | 9060041 | 9574287 | 9600509 | 8883790 | 9266096,6 | 315583,7 | 3,41 |
| **ACETYL PROPIONYL** | | 2375472 | 2390979 | 2347222 | 2321510 | 2328163 | 2352669,2 | 29953,0 | 1,27 |
| **ACETYL METHYL CARBINOL** | | 5917923 | 6014922 | 5516498 | 5478755 | 5727944 | 5731208,4 | 237276,0 | 4,14 |

| **DIACETYL** | | | | | | |
| --- | --- | --- | --- | --- | --- | --- |
| **CONTENT** | 0,99 | 9,9 | 49,5 | 99 | 198 | 396 |
| **AREA** | 100081 | 573877,6 | 1919734 | 2870967,4 | 5471363,5 | 9266096,6 |

| **ACETYL PROPIONYL** | | | | | | |
| --- | --- | --- | --- | --- | --- | --- |
| **CONTENT** | 0,957 | 9,57 | 47,85 | 95,7 | 191,4 | 382,8 |
| **AREA** | 13700,4 | 119447,6 | 453921,4 | 699386,2 | 1283593,5 | 2352669,2 |

| **ACETYL METHYL CARBINOL** | | | | | | |
| --- | --- | --- | --- | --- | --- | --- |
| **CONTENT** | 1 013 | 10,13 | 50,65 | 101,3 | 202,6 | 405,2 |
| **AREA** | 17898,2 | 140990,8 | 663957,2 | 1212858,8 | 2544811,25 | 5731208,4 |

Conclusion **:** The tests carried out show that the samples taken via the Gilian pumps are all linear. Requirements in terms of correlation coefficient values are to be weighted with regard to the fact that the samples were performed in the analytical laboratory, which by definition in an environment that is not VOCs free.

1. ***THE INFLUENCE OF HYGROMETRY***

To measure and try to model the impact of hygrometry on our samples, we used an ATIS^TM^ system distributed by SIGMA ALDRICH. This system (see opposite) has two distinct nitrogen inlets, both connected to a heat-controlled vaporization cell. The system is equipped with a dry nitrogen inlet and with an inlet for nitrogen humidified by a bubbler system. The injection of the microliter of standard solution is carried out through a septum located on the vaporization chamber. The relative humidity is adjusted by setting the proportions of wet and dry gas entering the vaporization cell.

**III-1** **Calibration of ATIS^TM^ System.**

| **WET (ml/min)** | **DRY (ml/min)** | **RH %** |
| --- | --- | --- |
| **0** | **50** | **8,3** |
| **10** | **40** | **23,5** |
| **20** | **30** | **36,3** |
| **30** | **20** | **49** |
| **40** | **10** | **66,3** |
| **50** | **0** | **77,5** |

**III-2 IMPACT OF HYGROMETRY ON SAMPLING OPERATIONS**

**III-2-1 Impact of hygrometry on a 5 minutes sampling time.**

Study of the impact of hygrometry variation on the preparation of our standard solutions : Entrapment of a 100ng/µl standard of each of the three target molecules (Diacetyl/Acetyl propionyl/Acetyl methyl carbinol) with a sampling time of 5 minutes.

- Flow rate: 50 ml/min

- Pumping time: 5 minutes

|  |  | **RH = 8,3%** | | |  |  |  |
| --- | --- | --- | --- | --- | --- | --- | --- |
|  | | **TUBE 1** | **TUBE 2** | **TUBE 3** | **AVERAGE** | **STD DEVIATION** | **CV %** |
| **DIACETYL** | | *4212136* | 4340551 | 4557696 | 4370127,67 | 174668,3 | 4,00 |
| **ACETYL PROPIONYL** | | *984430* | 1217784 | 1225090 | 1142434,67 | 136884,8 | 11,98 |
| **ACETYL METHYL CARBINOL** | | *1928577* | 2096219 | 1973682 | 1999492,67 | 86750,2 | 4,34 |
|  |  |  |  |  |  |  |  |
|  |  | **RH = 23,5%** | | |  |  |  |
|  | | **TUBE 1** | **TUBE 2** | **TUBE 3** | **AVERAGE** | **STD DEVIATION** | **CV %** |
| **DIACETYL** | | 4066795 | 4061997 | 4356467 | 4161753 | 168644,3 | 4,05 |
| **ACETYL PROPIONYL** | | 1103642 | 1105732 | 1135291 | 1114888,33 | 17700,1 | 1,59 |
| **ACETYL METHYL CARBINOL** | | 2049896 | 2047577 | 1902820 | 2000097,67 | 84252,9 | 4,21 |
|  |  |  |  |  |  |  |  |
|  |  | **RH = 36,3%** | | |  |  |  |
|  | | **TUBE 1** | **TUBE 2** | **TUBE 3** | **AVERAGE** | **STD DEVIATION** | **CV %** |
| **DIACETYL** | | 4103390 | 4313551 | 4198540 | 4205160,33 | 105236,8 | 2,50 |
| **ACETYL PROPIONYL** | | 1116031 | 1120981 | 1098826 | 1111946 | 11628,7 | 1,05 |
| **ACETYL METHYL CARBINOL** | | 2059872 | 2001779 | 1987677 | 2016442,67 | 38266,1 | 1,90 |
|  |  |  |  |  |  |  |  |
|  |  | **RH = 49%** | | |  |  |  |
|  | | **TUBE 1** | **TUBE 3** | **TUBE 2** | **AVERAGE** | **STD DEVIATION** | **CV %** |
| **DIACETYL** | | 4219599 | 4150552 | *2063100* | 4185075,5 | 48823,6 | 1,17 |
| **ACETYL PROPIONYL** | | 1112739 | 1128770 | *307623* | 1120754,5 | 11335,6 | 1,01 |
| **ACETYL METHYL CARBINOL** | | 1986661 | 2085071 | *770074* | 2035866 | 69586,4 | 3,42 |
|  |  |  |  |  |  |  |  |
|  |  | **RH = 66,3%** | | |  |  |  |
|  | | **TUBE 1** | **TUBE 2** | **TUBE 3** | **AVERAGE** | **STD DEVIATION** | **CV %** |
| **DIACETYL** | | 4455765 | 4571343 | 4378241 | 4468449,67 | 97173,9 | 2,17 |
| **ACETYL PROPIONYL** | | 1101276 | 1115225 | 1110044 | 1108848,33 | 7050,9 | 0,64 |
| **ACETYL METHYL CARBINOL** | | 1865398 | 1809313 | 1887973 | 1854228 | 40502,2 | 2,18 |
|  |  |  |  |  |  |  |  |
|  |  | **RH = 77,5%** | | |  |  |  |
|  | | **TUBE 1** | **TUBE 2** | **TUBE 3** | **AVERAGE** | **STD DEVIATION** | **CV %** |
| **DIACETYL** | | 4063033 | 4230673 | 4305157 | 4199621 | 124012,8 | 2,95 |
| **ACETYL PROPIONYL** | | 1106175 | 1124740 | 1102369 | 1111094,67 | 11969,5 | 1,08 |
| **ACETYL METHYL CARBINOL** | | 1487224 | 1537730 | 1477348 | 1500767,33 | 32389,2 | 2,16 |

(The values in italics were not taken into account for the average calculation)


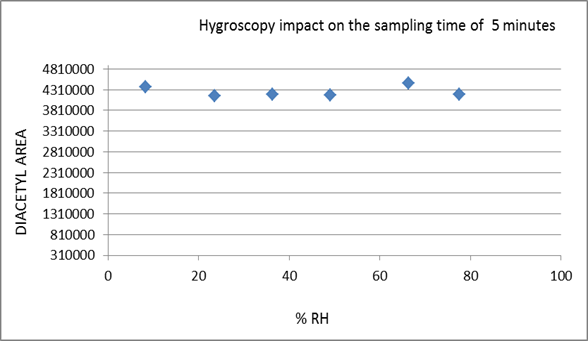


| **DIACETYL** | |
| --- | --- |
| **%RH** | **AREA** |
| 8,3 | 4370128 |
| 23,5 | 4161753 |
| 36,3 | 4205160 |
| 49 | 4185076 |
| 66,3 | 4468450 |
| 77,5 | 4199621 |


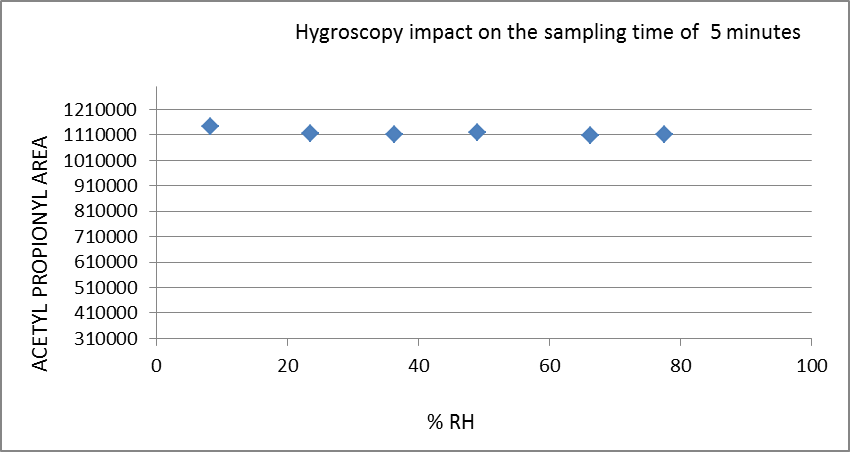


| **ACETYL PROPIONYL** | |
| --- | --- |
| **%RH** | **AREA** |
| 8,3 | 1142435 |
| 23,5 | 1114888 |
| 36,3 | 1111946 |
| 49 | 1120755 |
| 66,3 | 1108848 |
| 77,5 | 1111095 |


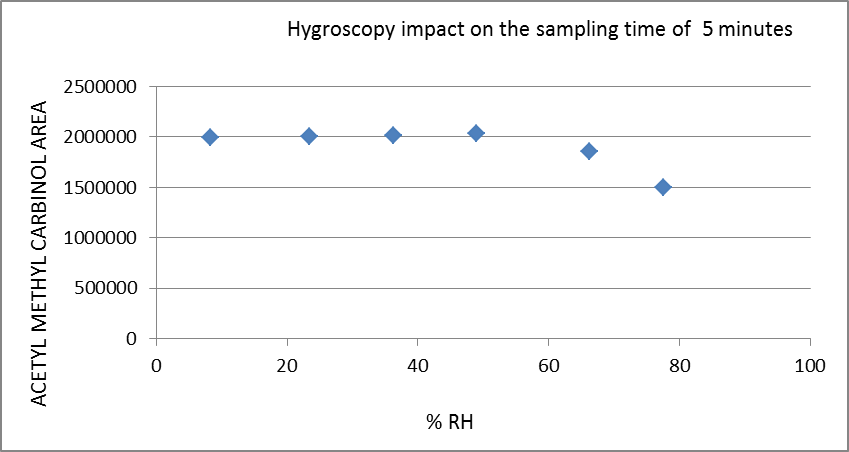


| **ACETYL METHYL CARBINOL** | |
| --- | --- |
| **%RH** | **AREA** |
| 8,3 | 1999492 |
| 23,5 | 2000097 |
| 36,3 | 2016442 |
| 49 | 2035866 |
| 66,3 | 1854228 |
| 77,5 | 1500767 |

**III-2-2 Impact of hygrometry on a 20 minutes sampling time.**

Study of the impact of hygrometry variation on the entrapment of a 100ng/µl standard

solution of each of the three target molecules (Diacetyl/Acetyl propionyl/Acetyl methyl carbinol).

- Flow rate: 50 ml/min
- Pumping time: 20 minutes

|  |  | | | **RH = 8,3%** | | | | |  | |  | |  | |  | | |
| --- | --- | --- | --- | --- | --- | --- | --- | --- | --- | --- | --- | --- | --- | --- | --- | --- | --- |
|  | | | | **TUBE 1** | **TUBE 2** | | **TUBE 3** | | **AVERAGE** | | **STD DEVIATION** | | **CV %** | |  | | |
| **DIACETYL** | | | | *4212136* | 4340551 | | 4557696 | | 4449123,5 | | 153544,7 | | 3,45 | |  | | |
| **ACETYL PROPIONYL** | | | | *984430* | 1217784 | | 1225090 | | 1221437 | | 5166,1 | | 0,42 | |  | | |
| **ACETYL METHYL CARBINOL** | | | | *1928577* | 2096219 | | 1973682 | | 2034950,5 | | 86646,7 | | 4,26 | |  | | |
|  |  | | |  |  | |  | |  | |  | |  | |  | | |
|  |  | | | **RH = 23,5%** | | | | |  | |  | |  | |  | | |
|  | | | | **TUBE 1** | **TUBE 2** | | **TUBE 3** | | **AVERAGE** | | **STD DEVIATION** | | **CV %** | |  | | |
| **DIACETYL** | | | | 4066795 | 4061997 | | 4356467 | | 4161753 | | 168644,3 | | 4,05 | |  | | |
| **ACETYL PROPIONYL** | | | | 1103642 | 1105732 | | 1135291 | | 1114888,3 | | 17700,1 | | 1,59 | |  | | |
| **ACETYL METHYL CARBINOL** | | | | 2049896 | 2047577 | | 1902820 | | 2000097,7 | | 84252,9 | | 4,21 | |  | | |
|  |  | | |  |  | |  | |  | |  | |  | |  | | |
|  |  | | | **RH = 36,3%** | | | | |  | |  | |  | |  | | |
|  | | | | **TUBE 1** | **TUBE 2** | | **TUBE 3** | | **AVERAGE** | | **STD DEVIATION** | | **CV %** | |  | | |
| **DIACETYL** | | | | 4103390 | 4313551 | | 4198540 | | 4205160,3 | | 105236,8 | | 2,50 | |  | | |
| **ACETYL PROPIONYL** | | | | 1116031 | 1120981 | | 1098826 | | 1111946 | | 11628,7 | | 1,05 | |  | | |
| **ACETYL METHYL CARBINOL** | | | | 2059872 | 2001779 | | 1987677 | | 2016442,7 | | 38266,1 | | 1,90 | |  | | |
|  |  | | |  |  | |  | |  | |  | |  | |  | | |
|  |  | | | **RH = 49%** | | | | |  | |  | |  | |  | | |
|  | | | | **TUBE 1** | **TUBE 3** | | **TUBE 2** | | **AVERAGE** | | **STD DEVIATION** | | **CV %** | |  | | |
| **DIACETYL** | | | | 4219599 | 4150552 | | *2063100* | | 4185075,5 | | 48823,6 | | 1,17 | |  | | |
| **ACETYL PROPIONYL** | | | | 1112739 | 1128770 | | *307623* | | 1120754,5 | | 11335,6 | | 1,01 | |  | | |
| **ACETYL METHYL CARBINOL** | | | | 1986661 | 2085071 | | *770074* | | 2035866 | | 69586,4 | | 3,42 | |  | | |
|  |  | | |  |  | |  | |  | |  | |  | |  | | |
|  |  | | | **RH = 66,3%** | | | | |  | |  | |  | |  | | |
|  | | | | **TUBE 1** | **TUBE 2** | | **TUBE 3** | | **AVERAGE** | | **STD DEVIATION** | | **CV %** | |  | | |
| **DIACETYL** | | | | 4455765 | 4571343 | | 4378241 | | 4468449,7 | | 97173,9 | | 2,17 | |  | | |
| **ACETYL PROPIONYL** | | | | 1101276 | 1115225 | | 1110044 | | 1108848,3 | | 7050,9 | | 0,64 | |  | | |
| **ACETYL METHYL CARBINOL** | | | | 1865398 | 1809313 | | 1887973 | | 1854228,0 | | 40502,2 | | 2,18 | |  | | |
|  |  | | |  |  | |  | |  | |  | |  | |  | | |
|  |  | | | **RH = 77,5%** | | | | |  | |  | |  | |  | | |
|  | | | | **TUBE 1** | **TUBE 2** | | **TUBE 3** | | **AVERAGE** | | **STD DEVIATION** | | **CV %** | |  | | |
| **DIACETYL** | | | | 4063033 | 4230673 | | 4305157 | | 4199621 | | 124012,8 | | 2,95 | |  | | |
| **ACETYL PROPIONYL** | | | | 1106175 | 1124740 | | 1102369 | | 1111094,7 | | 11969,5 | | 1,08 | |  | | |
| **ACETYL METHYL CARBINOL** | | | | 1487224 | 1537730 | | 1477348 | | 1500767,3 | | 32389,2 | | 2,16 | |  | | |
|  | |  |  | | |  | |  | |  | |  | |  | |  |  |
|  | |  |  | | |  | |  | |  | |  | |  | |  |  |
|  | |  | 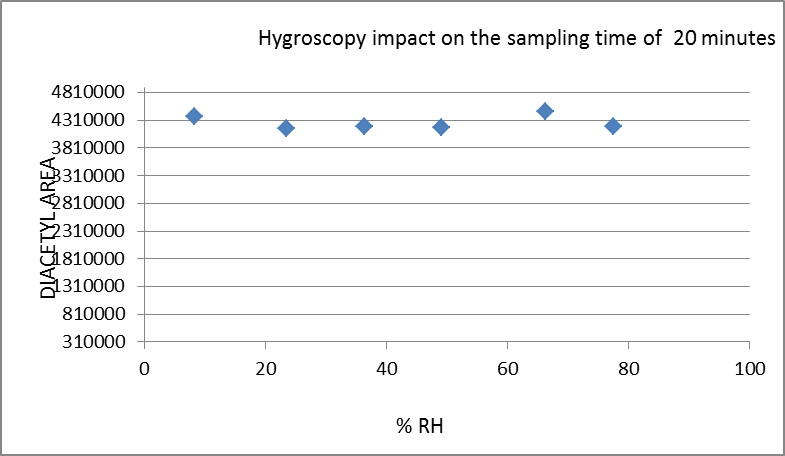 | | |  | |  | |  | |  | |  | |  |  |
| **DIACETYL** | | |  | | |  | |  | |  | |  | |  | |  |  |
| **%RH** | | **AREA** |  | | |  | |  | |  | |  | |  | |  |  |
| 8,3 | | 3651273,67 |  | | |  | |  | |  | |  | |  | |  |  |
| 23,5 | | 3520706,67 |  | | |  | |  | |  | |  | |  | |  |  |
| 36,3 | | 3767909,00 |  | | |  | |  | |  | |  | |  | |  |  |
| 49 | | 3776356,00 |  | | |  | |  | |  | |  | |  | |  |  |
| 66,3 | | 3984498,33 |  | | |  | |  | |  | |  | |  | |  |  |
| 77,5 | | 3847584,33 |  | | |  | |  | |  | |  | |  | |  |  |
|  | |  |  | | |  | |  | |  | |  | |  | |  |  |
|  | |  |  | | |  | |  | |  | |  | |  | |  |  |
|  | |  |  | | |  | |  | |  | |  | |  | |  |  |
|  | |  |  | | |  | |  | |  | |  | |  | |  |  |
|  | |  | 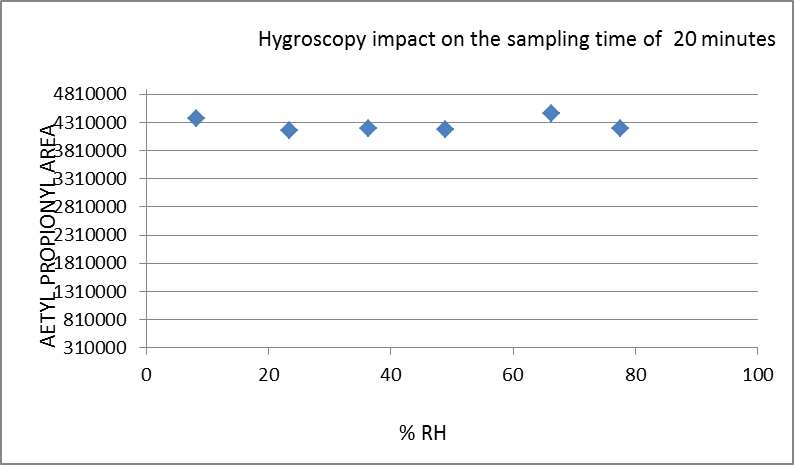 | | |  | |  | |  | |  | |  | |  |  |
|  | |  |  | | | | | | | | | | | | | | |
|  | |  |
| **ACETYL PROPIONYL** | | |
| **%RH** | | **AREA** |
| 8,3 | | 9060143,3 |
| 23,5 | | 8545083,3 |
| 36,3 | | 9181873,3 |
| 49 | | 928081,5 |
| 66,3 | | 907963 |
| 77,5 | | 829769 |
|  | |  |
|  | |  |
|  | |  |
|  | |  |
|  | |  |
|  | |  | 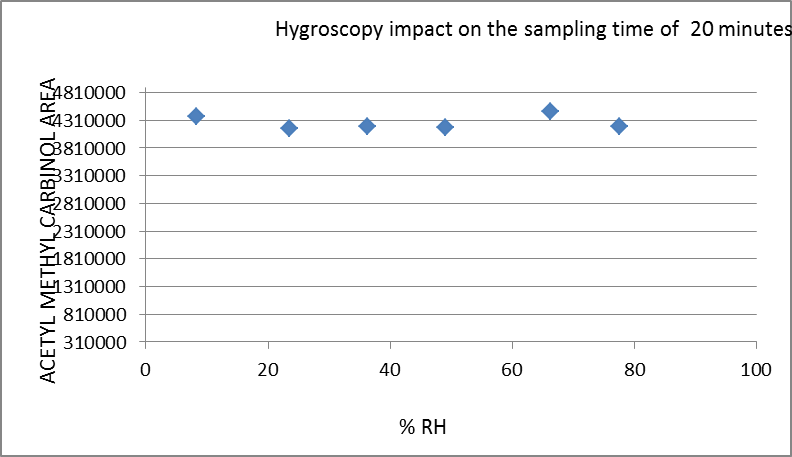 | | | | | | | | | | | | | | |
|  | |  |
| **ACETYL MET CARBINOL** | | |
| **%RH** | | **AREA** |
| 8,3 | | 1716840,7 |
| 23,5 | | 1641207,7 |
| 36,3 | | 1741810,3 |
| 49 | | 1947746,5 |
| 66,3 | | 1735725 |
| 77,5 | | 1645306,7 |
|  | |  |
|  | |  |
|  | |  |
|  | |  |
|  | |  |

(The values in italics were not taken into account for the average calculation).

**III-2-3 Impact of hygrometry on a 70 minutes sampling time.**

Study of the impact of hygrometry variation on the entrapment of a 100ng/µl standard

solution of each of the three target molecules (Diacetyl/Acetyl propionyl/Acetyl methyl carbinol).

- Flow rate: 50 ml/min
- Pumping time: 70 minutes.

|  |  |  | **RH = 23,5%** | | |  |  |  |
| --- | --- | --- | --- | --- | --- | --- | --- | --- |
|  | | | **TUBE 1** | **TUBE 2** | **TUBE 3** | **AVERAGE** | **STD DEVIATION** | **CV %** |
| **DIACETYL** | | | 1358557 | 1071973 | 1122052 | 1184194 | 153064,8 | 12,93 |
| **ACETYL PROPIONYL** | | | 189879 | 177390 | 189550 | 185606,333 | 7117,5 | 3,83 |
| **ACETYL METHYL CARBINOL** | | | 1372143 | 1410137 | 1470956 | 1417745,33 | 49843,9 | 3,52 |
|  |  |  |  |  |  |  |  |  |
|  |  |  | **RH = 36,3%** | | |  |  |  |
|  | | | **TUBE 1** | **TUBE 2** | **TUBE 3** | **AVERAGE** | **STD DEVIATION** | **CV %** |
| **DIACETYL** | | | 1239029 | 1260110 | 1237623 | 1245587,33 | 12596,6 | 1,01 |
| **ACETYL PROPIONYL** | | | 220508 | 230387 | 222022 | 224305,667 | 5320,7 | 2,37 |
| **ACETYL METHYL CARBINOL** | | | 1405195 | 1495639 | 1405740 | 1435524,67 | 52061,3 | 3,63 |
|  |  |  |  |  |  |  |  |  |
|  |  |  | **RH = 49%** | | |  |  |  |
|  | | | **TUBE 1** | **TUBE 3** | **TUBE 2** | **AVERAGE** | **STD DEVIATION** | **CV %** |
| **DIACETYL** | | | 1309019 | 1295047 | 1243688 | 1282584,67 | 34402,3 | 2,68 |
| **ACETYL PROPIONYL** | | | 239376 | 235308 | 232790 | 235824,667 | 3323,3 | 1,41 |
| **ACETYL METHYL CARBINOL** | | | 1482839 | 1465127 | 1499178 | 1482381,33 | 17030,1 | 1,15 |
|  |  |  |  |  |  |  |  |  |
|  |  |  | **RH = 66,3%** | | |  |  |  |
|  | | | **TUBE 1** | **TUBE 2** | **TUBE 3** | **AVERAGE** | **STD DEVIATION** | **CV %** |
| **DIACETYL** | | | 1341655 | 1252417 | 1209097 | 1267723 | 67591,5 | 5,33 |
| **ACETYL PROPIONYL** | | | 220041 | 209618 | 198232 | 209297 | 10908,0 | 5,21 |
| **ACETYL METHYL CARBINOL** | | | 1506614 | 1432194 | 1367815 | 1435541 | 69460,0 | 4,84 |
|  |  |  |  |  |  |  |  |  |
|  |  |  | **RH = 77,5%** | | |  |  |  |
|  | | | **TUBE 1** | **TUBE 2** | **TUBE 3** | **AVERAGE** | **STD DEVIATION** | **CV %** |
| **DIACETYL** | | | 1694803 | 1597134 | 1645333 | 1645756,67 | 48835,9 | 2,97 |
| **ACETYL PROPIONYL** | | | 201546 | 207205 | 208776 | 205842,333 | 3802,7 | 1,85 |
| **ACETYL METHYL CARBINOL** | | | 1066421 | 1219774 | 1146483 | 1144226 | 76701,4 | 6,70 |

|  |  | |  | | --- | |  |  |  |  |  |  |
| --- | --- | --- | --- | --- | --- | --- | --- | --- | --- |
|  |  |  |  |  |  |  |  |  |
|  |  | 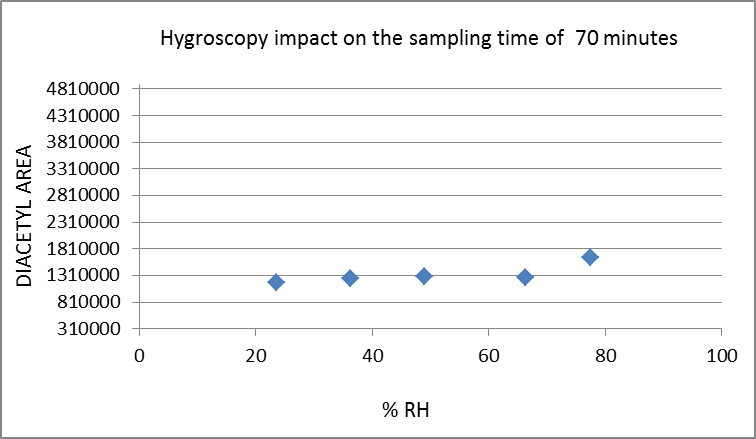 |  |  |  |  |  |  |
| **DIACETYL** | |  |  |  |  |  |  |  |
| **% RH** | **AREA** |  |  |  |  |  |  |  |
| 23,5 | 1184194 |  |  |  |  |  |  |  |
| 36,3 | 1245587,3 |  |  |  |  |  |  |  |
| 49 | 1282584,7 |  |  |  |  |  |  |  |
| 66,3 | 1267723 |  |  |  |  |  |  |  |
| 77,5 | 1645756,7 |  |  |  |  |  |  |  |
|  |  |  |  |  |  |  |  |  |
|  |  |  |  |  |  |  |  |  |
|  |  |  |  |  |  |  |  |  |
|  |  |  |  |  |  |  |  |  |
|  |  |  |  |  |  |  |  |  |
|  |  | 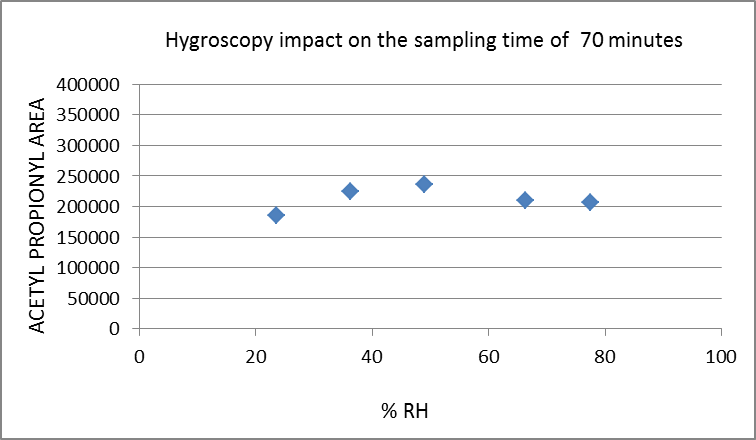 | | | | | | |
|  |  |
| **ACETYL PROPIONYL** | |
| **% RH** | **AREA** |
| 23,5 | 185606,3 |
| 36,3 | 224305,7 |
| 49 | 235824,7 |
| 66,3 | 209297 |
| 77,5 | 205842 |
|  |  |
|  |  |
|  |  |
|  |  |
|  |  |
|  |  |  |  |  |  |  |  |  |
|  |  | 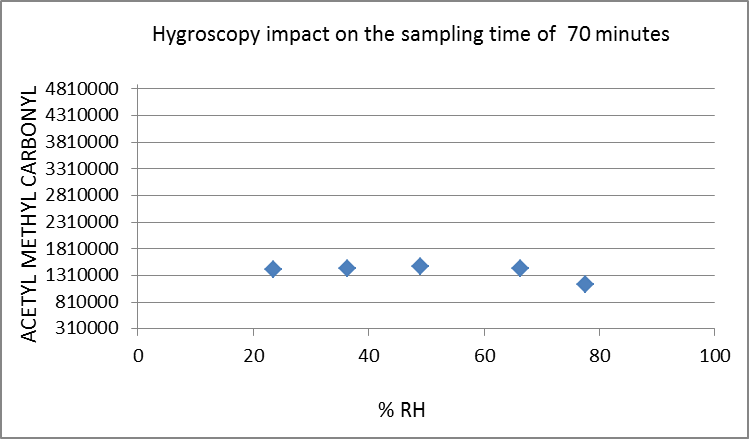 | | | | | | |
|  |  |
| **ACETYL METHYL CARBINOL** | |
| **% RH** | **AREA** |
| 23,5 | 1417745,3 |
| 36,3 | 1435524,7 |
| 49 | 1482381,3 |
| 66,3 | 1435541 |
| 77,5 | 1144226 |
|  |  |
|  |  |
|  |  |
|  |  |
|  |  |
|  |  |

Conclusions : These tests emphasize hygrometry low interference on the quantities

of target molecules sampled by our devices. These results are explained by the fact that

TENAX is a low-absorbent and hydrophobic material and not very sensitive to hygrometry

variations.

| **Name** | **use range** | **Water retention**  Weak  Medium  Strong  Sample   |  | | --- | |  |  |  |  |  |  |
| --- | --- | --- | --- | --- | --- | --- | --- | --- | --- |
| Wool Quartz / Silica beads | C30 - C40 |  |  |  |  |  |  |  |
| Tenax® TA | C7 - C30 |  |  |  |  |  |  |  |
| Carbograph ^TM^ 2TD | C8 - C20 |  |  |  |  |  |  |  |
| Carbograph ^TM^ 1TD | C5/6 - C14 |  |  |  |  |  |  |  |
| Carbopack ^TM^ X | C3/4 - C6/7 |  |  |  |  |  |  |  |
| UniCarb ^TM^ | C3 - C8 |  |  |  |  |  |  |  |

1. ***SEALING OF THE SAMPLING DEVICES***

Exposure of 6 sealed TENAX tubes to an atmosphere of test under the following conditions:

**-** Concentration of exposure: solution corresponding to 400 ng/μl of each required compounds

(norm 2 VL).

**-** Time : 4 hours

**-** Relative humidity : (50 +/-5) %

**-** Temperature : (20 +/-2) °C.

Analyzes by TD-GC/MS of these tubes in order to detect and quantify the possible escapes according to standard NF EN 1076.

The maximum escape, i.e. the maximum mass taken of component to quantify must be lower than m_a,lt_, calculated according to the following equation :

m_a,lt LV,st_ ^-3^ = 1/3 ( 0.1 ρ*240*0.01*10).

m_a,lt_: is the maximum mass uptake of analyte in a leak test performed on a sealed sampler used for making

measurements for comparison with a long-term limit value ( en mg)

ρ_LV,st_: is the long-term limit value of the substance given as volume concentration, expressed in mg/m3.

- ρ_LV,st (diacetyl)_ = (VLppm _diacetyl_*MM _diacetyl_)/24.46 = (0.005*86)/24.46 = 0.0176 mg/m3, soit 17580

ng/m3.

- ρ_LV,st (acetyl propionyl)_ = (VLppm _acetyl propionyl_*MM _acetyl propionyl_)/24.46 = (0.005*100)/24.46 = 0.0204

mg/m3, soit 20441 ng/m3.

- ρ_LV,st(acetyl methyl carbinol)_ = (VLppm _acetyl methyl carbinol_*MM _acetyl methyl carbinol_)/24.46 = (0.005*88)/24.46 =

0.0180 mg/m3, soit 17988 ng/m3.

- m_a,lt(diacetyl)_ = 1/3 ( 0.1 ρ_LV,st(diacetyl)_ *240*0.01*10^-3^ ) = 1.408 10^-6^ mg soit 1.408ng.
- m_a,lt(acetyl propionyl)_ = 1/3 ( 0.1 ρ_LV,st(acetyl propionyl)_ *240*0.01*10^-3^ ) = 1.632 10^-6^ mg soit 1.632ng.
- m_a,lt(acetyl methyl carbinol)_ = 1/3 ( 0.1 ρ_LV,st(acetyl methyl carbinol)_ *240*0.01*10^-3^ ) = 1.44 10^-6^ mg soit 1.44ng.

240 : is the reference period, in minutes,

0.01 : Is the nominal minimal flow for the sampling devices which resort to adsorption on a solid and

with a thermal desorption, in (l/min)

10^-3^ : Multiplicative factor to convert the nominal minimal flow of l/min in m^3^/min.

1/3  : Factor applied to calculate the maximum permitted leakage.

|  | **TUBE 1** | **TUBE 2** | **TUBE 3** | **TUBE 4** | **TUBE 5** | **TUBE 6** | **AVERAGE** | **STD DEVIATION** | **CV %** | **% Adsorbed** | **Adsorbed mass (µg)** |
| --- | --- | --- | --- | --- | --- | --- | --- | --- | --- | --- | --- |
| **DIACETYL** | 45864 | 49946 | 46567 | 48186 | 37163 | 47591 | 45886,17 | 4499,7 | 9,81 | 0,31 | 1 245 |
| **ACETYL PROPIONYL** | 7464 | 7731 | 7605 | 7639 | 7665 | 7261 | 7560,83 | 171,5 | 2,27 | 0,19 | 0,759 |
| **ACETYL METHYL CARBINOL** | 14359 | 12936 | 14256 | 14209 | 15043 | 9008 | 13301,83 | 2211,5 | 16,63 | 0,22 | 0,888 |

Conclusion : The leak test was carried out with a solution very largely higher than that recommended by standard, NF EN 1076 (2*VL). In spite of the extreme conditions of the test, the maximum masses adsorbed for three target molecules, remain lower than the calculated limit values.

***V- BREAKTHROUGH OF SAMPLING DEVICES WITH A FIXED FLOW RATE (50 ml/min) ANALYSIS***

Definition: Volume of air of known composition likely to pass through the tube before the outgoing

vapor concentration reaches 5% of the concentration applied for test (EN1076).

The breakthrough volume is measured by sampling a test atmosphere under the following conditions:

- Concentration: standard solution of 100ng/µl
- Flow rate: 50 ml/min (flow rate recommended by the standard, AFNOR NF EN 1076, for

devices which use adsorption on solids, thermal desorption and analysis of the desorbate)

- Relative humidity: 80%
- Temperature: (20±2)°C.

The sampling is carried out by injection of one microliter of the 100 ng/µl standard solution

into the entrapment system. A 50 ml/min fixed flow rate for pumping provides the adsorption

of our target molecules on the 2 Tenax tubes assembled in series.

| **5 minutes** | |  | **TUBE N°1** | **TUBE N°2** | **BREAKTHROUGH** | **Average** |
| --- | --- | --- | --- | --- | --- | --- |
| **DIACETYL** | | **TEST 1** | 1023558 | 41595 | 4,1 | 4,7 |
|  |  | **TEST 2** | 1029686 | 46637 | 4,5 |  |
|  |  | **TEST 3** | 969476 | 54004 | 5,6 |  |
| **ACETYL PROPIONYL** | | **TEST 1** | 131397 | 1586 | 1,2 | 1,2 |
|  |  | **TEST 2** | 122748 | 1290 | 1,1 |  |
|  |  | **TEST 3** | 120280 | 1769 | 1,5 |  |
| **ACETYL METHYL CARBINOL** | | **TEST 1** | 1529236 | 4461 | 0,3 | 0,3 |
|  |  | **TEST 2** | 1480299 | 4903 | 0,3 |  |
|  |  | **TEST 3** | 1452555 | 5096 | 0,4 |  |
|  |  |  |  |  |  |  |
| **15 minutes** | |  | **TUBE N°1** | **TUBE N°2** | **BREAKTHROUGH** | **Average** |
| **DIACETYL** | | **TEST 1** | 1068125 | 63129 | 5,9 | 4,8 |
|  |  | **TEST 2** | 1017343 | 40365 | 4,0 |  |
|  |  | **TEST 3** | 1133762 | 51681 | 4,6 |  |
| **ACETYL PROPIONYL** | | **TEST 1** | 126780 | 1727 | 1,4 | 1,3 |
|  |  | **TEST 2** | 125519 | 1470 | 1,2 |  |
|  |  | **TEST 3** | 130207 | 1749 | 1,3 |  |
| **ACETYL METHYL CARBINOL** | | **TEST 1** | 1511983 | 9447 | 0,6 | 0,5 |
|  |  | **TEST 2** | 1498754 | 7357 | 0,5 |  |
|  |  | **TEST 3** | 1488761 | 5171 | 0,3 |  |
|  |  |  |  |  |  |  |
| **30 minutes** | |  | **TUBE N°1** | **TUBE N°2** | **BREAKTHROUGH** | **Average** |
| **DIACETYL** | | **TEST 1** | 1036727 | 81729 | 7,9 | 6,9 |
|  |  | **TEST 2** | 1162159 | 63692 | 5,5 |  |
|  |  | **TEST 3** | 1131302 | 59807 | 7,2 |  |
| **ACETYL PROPIONYL** | | **TEST 1** | 126415 | 2043 | 1,6 | 1,4 |
|  |  | **TEST 2** | 133496 | 1484 | 1,1 |  |
|  |  | **TEST 3** | 126366 | 2667 | 1,6 |  |
| **ACETYL METHYL CARBINOL** | | **TEST 1** | 1452902 | 7359 | 0,5 | 0,5 |
|  |  | **TEST 2** | 1459914 | 7824 | 0,5 |  |
|  |  | **TEST 3** | 1466601 | 9939 | 0,5 |  |
|  | |  |  |  |  |  |

| **60 minutes** |  | **TUBE N°1** | **TUBE N°2** | **BREAKTHROUGH** | **Average** |
| --- | --- | --- | --- | --- | --- |
| **DIACETYL** | **TEST 1** | 1108194 | 67949 | 6,1 | 13,3 |
|  | **TEST 2** | 1052621 | 148330 | 14,1 |  |
|  | **TEST 3** | 1123289 | 221334 | 19,7 |  |
| **ACETYL PROPIONYL** | **TEST 1** | 126302 | 1177 | 0,9 | 2,5 |
|  | **TEST 2** | 123583 | 3711 | 3,0 |  |
|  | **TEST 3** | 134667 | 4669 | 3,5 |  |
| **ACETYL METHYL CARBINOL** | **TEST 1** | 1375171 | 10284 | 0,7 | 0,8 |
|  | **TEST 2** | 1321498 | 12405 | 0,9 |  |
|  | **TEST 3** | 1372664 | 10629 | 0,8 |  |

Conclusion: These results enable us to envisage sampling on sites up to 30 minutes with the restriction

that the sampling takes place in conditions comparable to the test, i.e. in an environment almost entirely

free of other VOCs.

***VI- STORAGE STUDY***

The results obtained during these tests will provide us with important information because

there will certainly be significant delays between the taking of samples in the subsidiaries and

their analyses in Bar-Sur-Loup. These tests will therefore enable us to estimate a maximum storage

time for our sample tubes before analysis.

In accordance with the standard, NF X 43-267, storage is studied at room temperature (20-25°C)

and with a relative humidity of approximately 50%. The study is carried out on two concentrations:

high at 400 ng/µl and low at 10 ng/µl.

60 tubes are loaded, 30 with the 400 ng/µl solution and 30 with the 10 ng/µl solution. Then 6 groups

of 6 tubes for each concentration are produced, each group being stored under the same conditions

and analyzed according to the definite schedule: T_0_, T_3 days_, T_7 days_, T_10 days_ and T_15 days_.

| **10ng T0 : 01/06/2013** | | **TUBE 1** | **TUBE 2** | **TUBE 3** | **TUBE 4** | **TUBE 5** | **TUBE 6** | **Average** | **STD DEVIATION** | **% Analytical Recovery** |
| --- | --- | --- | --- | --- | --- | --- | --- | --- | --- | --- |
| **DIACETYL** | | 430726 | 540147 | 521759 | 462677 | 452973 | 437733 | 4743358,333 | 45637,6 | 9,6 |
| **ACETYL PROPIONYL** | | 114238 | 132205 | 125007 | 112178 | 113368 | 116577 | 1189288,333 | 7965,3 | 6,7 |
| **ACETYL METHYL CARBINOL** | | 181675 | 203667 | 181456 | 149472 | 175756 | 184926 | 179492 | 17537,0 | 9,8 |
|  |  |  |  |  |  |  |  |  |  |  |
|  |  |  |  |  |  |  |  |  |  |  |
| **400ng T0 : 01/06/2013** | | **TUBE 1** | **TUBE 2** | **TUBE 3** | **TUBE 4** | **TUBE 5** | **TUBE 6** | **Average** | **STD DEVIATION** | **% Analytical Recovery** |
| **DIACETYL** | | 11555537 | 12227214 | 11534615 | 11979104 | 11612103 | 12033846 | 11823736,5 | 293731,2 | 2,5 |
| **ACETYL PROPIONYL** | | 3204839 | 3371435 | 3236242 | 3437539 | 3355510 | 3386897 | 3332077 | 91213,5 | 2,7 |
| **ACETYL METHYL CARBINOL** | | 5692175 | 5958117 | 5520048 | 6095770 | 5913685 | 6173091 | 5892147,667 | 246436,3 | 4,2 |
|  |  |  |  |  |  |  |  |  |  |  |
|  |  |  |  |  |  |  |  |  |  |  |
| **10ng T3 days : 03/07/2013** | | **TUBE 1** | **TUBE 2** | **TUBE 3** | **TUBE 4** | **TUBE 5** | **TUBE 6** | **Average** | **STD DEVIATION** | **% Analytical Recovery** |
| **DIACETYL** | | 573830 | 526302 | 525566 | 506750 | 551637 | *328014* | 536817 | 26128,4 | 4,9 |
| **ACETYL PROPIONYL** | | 135955 | 128302 | 126677 | 116974 | 129083 | *33899* | 127398,2 | 6820,2 | 5,4 |
| **ACETYL METHYL CARBINOL** | | 134912 | 133643 | 108225 | 129502 | 141824 | *32756* | 129621,2 | 12756,2 | 9,8 |
|  |  |  |  |  |  |  |  |  |  |  |
|  |  |  |  |  |  |  |  |  |  |  |
| **400ng T3 days : 03/07/2013** | | **TUBE 1** | **TUBE 2** | **TUBE 3** | **TUBE 4** | **TUBE 5** | **TUBE 6** | **Average** | **STD DEVIATION** | **% Analytical Recovery** |
| **DIACETYL** | | 13640064 | 13664910 | 13610044 | 14132201 | 14009467 | 13805327 | 13810335,5 | 216117,3 | 1,6 |
| **ACETYL PROPIONYL** | | 3512604 | 3601228 | 3572595 | 3647411 | 3699990 | 3750848 | 3630779,333 | 86841,6 | 2,4 |
| **ACETYL METHYL CARBINOL** | | 5272067 | 5631727 | 5505994 | 5423277 | 5806965 | 5972113 | 5602023,833 | 256862,9 | 4,6 |
|  |  |  |  |  |  |  |  |  |  |  |
|  |  |  |  |  |  |  |  |  |  |  |
| **10ng T5 days : 05/07/2013** | | **TUBE 1** | **TUBE 2** | **TUBE 3** | **TUBE 4** | **TUBE 5** | **TUBE 6** | **Average** | **STD DEVIATION** | **% Analytical Recovery** |
| **DIACETYL** | | 570022 | 653013 | 580693 | 541586 | 613747 | 597482 | 592757,2 | 38404,6 | 6,5 |
| **ACETYL PROPIONYL** | | 125154 | 135824 | 129700 | 124494 | 129447 | 134250 | 129811,5 | 4603,6 | 3,5 |
| **ACETYL METHYL CARBINOL** | | 115460 | 135239 | 114614 | 117717 | 83157 | 131606 | 116298,8 | 18422,7 | 15,8 |
|  |  |  |  |  |  |  |  |  |  |  |
|  |  |  |  |  |  |  |  |  |  |  |
| **400ng T5 days : 05/07/2013** | | **TUBE 1** | **TUBE 2** | **TUBE 3** | **TUBE 4** | **TUBE 5** | **TUBE 6** | **Average** | **STD DEVIATION** | **% Analytical Recovery** |
| **DIACETYL** | | 14031741 | 14291519 | 14057517 | 14455896 | 13951141 | 13468864 | 14042780 | 337832 | 2,4 |
| **ACETYL PROPIONYL** | | 3540550 | 3681928 | 3529314 | 3687025 | 3369859 | 3551087 | 3559961 | 117094 | 3,3 |
| **ACETYL METHYL CARBINOL** | | 5267174 | 5301013 | 5138312 | 5388593 | 4724717 | 5625515 | 5240887 | 300338 | 5,7 |
|  |  |  |  |  |  |  |  |  |  |  |
|  |  |  |  |  |  |  |  |  |  |  |
| **10ng T9 days : 09/07/2013** | | **TUBE 1** | **TUBE 2** | **TUBE 3** | **TUBE 4** | **TUBE 5** | **TUBE 6** | **Average** | **STD DEVIATION** | **% Analytical Recovery** |
| **DIACETYL** | | 569245 | 585126 | 569654 | 544121 | 593482 | 581759 | 573898 | 17307 | 3,0 |
| **ACETYL PROPIONYL** | | 120095 | 122084 | 121612 | 122299 | 122510 | 120290 | 121482 | 1044 | 0,9 |
| **ACETYL METHYL CARBINOL** | | 86321 | 69523 | 87303 | 101502 | 82259 | 84363 | 85212 | 10262 | 12,0 |
|  |  |  |  |  |  |  |  |  |  |  |
|
| **400ng T9 jours le 09/07/2013** | | **TUBE 1** | **TUBE 2** | **TUBE 3** | **TUBE 4** | **TUBE 5** | **TUBE 6** | **Average** | **STD DEVIATION** | **% Analytical Recovery** |
| **DIACETYL** | | 14305129 | 14509890 | 13467459 | 13487618 | 13730027 | 14378168 | 13979715 | 471731,8 | 3,4 |
| **ACETYL PROPIONYL** | | 3565279 | 3536981 | 3471135 | 3280692 | 3505492 | 3575113 | 3489115 | 109104,9 | 3,1 |
| **ACETYL METHYL CARBINOL** | | 4846370 | 4400164 | 4739478 | 4148372 | 4564956 | 4561504 | 4543474 | 248029,6 | 5,5 |
|  |  |  |  |  |  |  |  |  |  |  |
|  |  |  |  |  |  |  |  |  |  |  |
| **10ng T16 jours le 16/07/2013** | | **TUBE 1** | **TUBE 2** | **TUBE 3** | **TUBE 4** | **TUBE 5** | **TUBE 6** | **Average** | **STD DEVIATION** | **% Analytical Recovery** |
| **DIACETYL** | | 695497 | 732875 | 736642 | 697104 | 703884 | 848803 | 735801 | 58171 | 7,9 |
| **ACETYL PROPIONYL** | | 140439 | 153031 | 149260 | 154188 | 156262 | 158348 | 151921 | 6410 | 4,2 |
| **ACETYL METHYL CARBINOL** | | 65477 | 85553 | 81660 | 89023 | 91822 | 78414 | 81992 | 9428 | 11,5 |
|  |  |  |  |  |  |  |  |  |  |  |
|
| **400ng T16 jours le 16/07/2013** | | **TUBE 1** | **TUBE 2** | **TUBE 3** | **TUBE 4** | **TUBE 5** | **TUBE 6** | **Average** | **STD DEVIATION** | **% Analytical Recovery** |
| **DIACETYL** | | 14853440 | 14818899 | 14635966 | 14257341 | 14237277 | 14031134 | 14472342,83 | 343003,1 | 2,4 |
| **ACETYL PROPIONYL** | | 4383663 | 4210119 | 4472117 | 4258084 | 4213803 | 4169367 | 4284525,5 | 117913,1 | 2,8 |
| **ACETYL METHYL CARBINOL** | | 5712133 | 5354148 | 6138261 | 5351885 | 5246800 | 5309563 | 5518798,333 | 344628,7 | 6,2 |

(The values in italics were not taken into account for the average calculation).

| **Study of the Conservation (Tubes 10ng)** | | | | | |
| --- | --- | --- | --- | --- | --- |
| **Number of days of conservation** | **0** | **3** | **5** | **9** | **16** |
| **DIACETYL** | 592757,2 | 536817 | 592757 | 573898 | 625431 |
| **ACETYL PROPIONYL** | 129811,5 | 127398,2 | 129812 | 121482 | 129133 |
| **ACETYL METHYL CARBINOL** | 116298,8 | 129621,2 | 116299 | 85211,8 | 69692,8 |


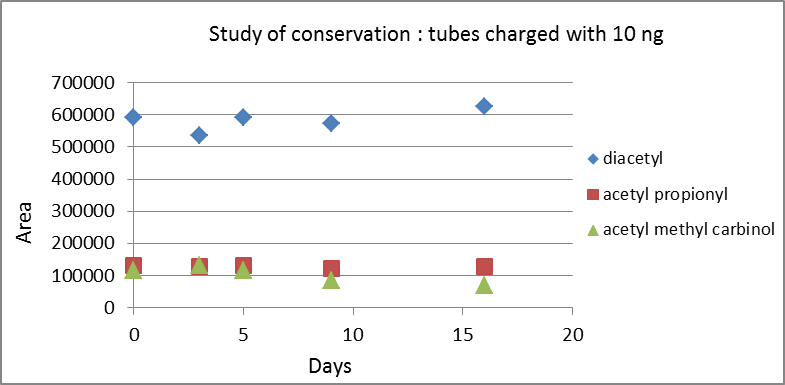


| **Study of the Conservation (Tubes 400ng)** | | | | | |
| --- | --- | --- | --- | --- | --- |
| **Number of days of conservation** | **0** | **3** | **5** | **9** | **16** |
| **DIACETYL** | 13823737 | 13810336 | 14042780 | 1,4E+07 | 1,4E+07 |
| **ACETYL PROPIONYL** | 3332077 | 3630779 | 3559960,5 | 3489115 | 4284526 |
| **ACETYL METHYL CARBINOL** | 5892148 | 5602024 | 5240887,3 | 4543474 | 5518798 |

Conclusion : According to the standard, NF EN 1076, the average value of recovery after storage

must not deviate by more than 10% of the value before storage (T0).

However, considering the accuracy of the method, we can estimate that these tests

reveal an acceptable storage of our samples over periods that may reach up to 16

days before analysis. In particular, we do not detect significant deterioration in our

three target molecules over this period.

1. ***SAMPLING TEST IN A CLOSED CHAMBER***

To study the influence of the concentration of the solution examined at a constant sampling

volume, 10 ml of solutions with increasing concentrations from 1 to 100 ng/µl (0.028 at

2.8%) are placed successively in a sealed enclosure with a volume of approximately 5 liters.

An equal volume of each atmosphere is sampled (1.5 L) and is analyzed:


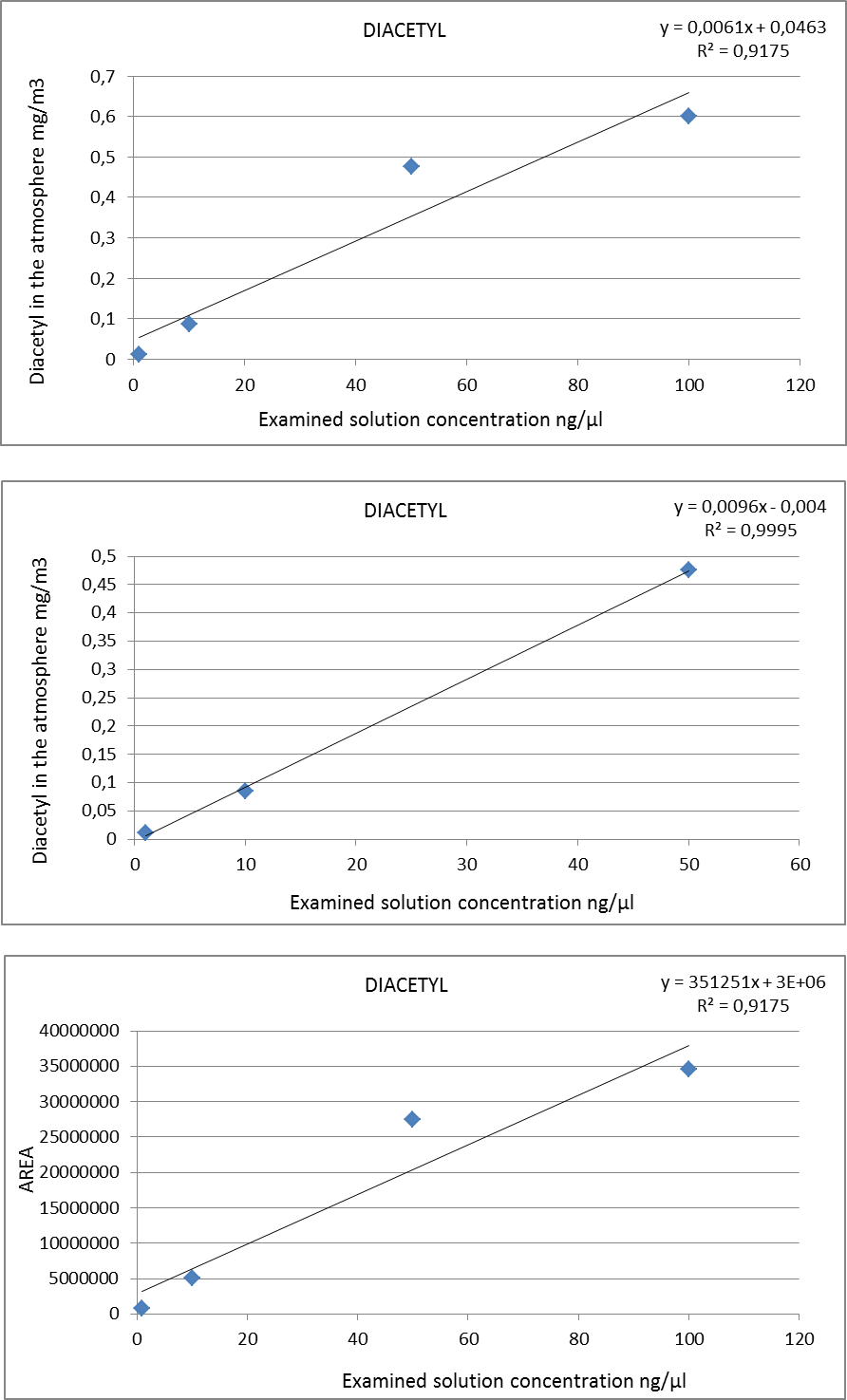


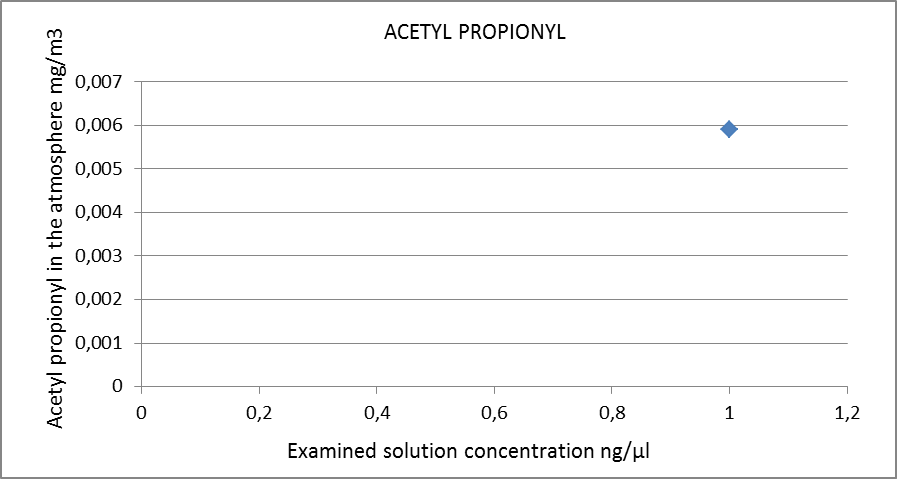


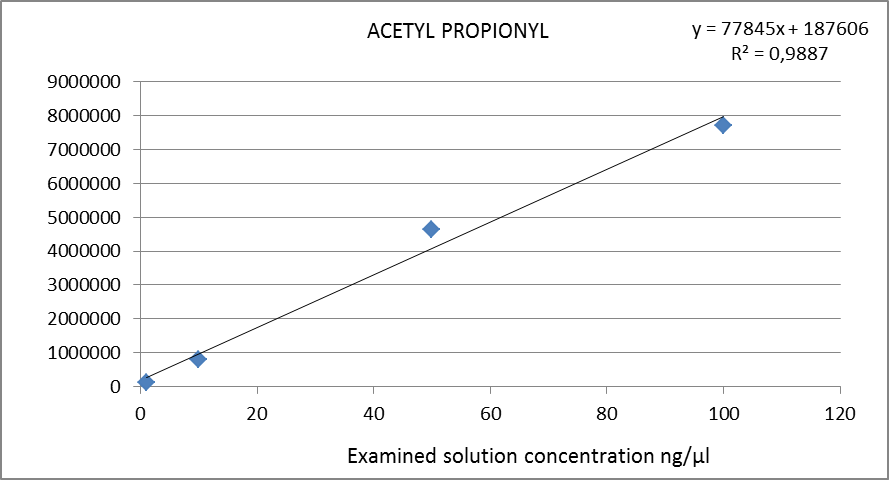


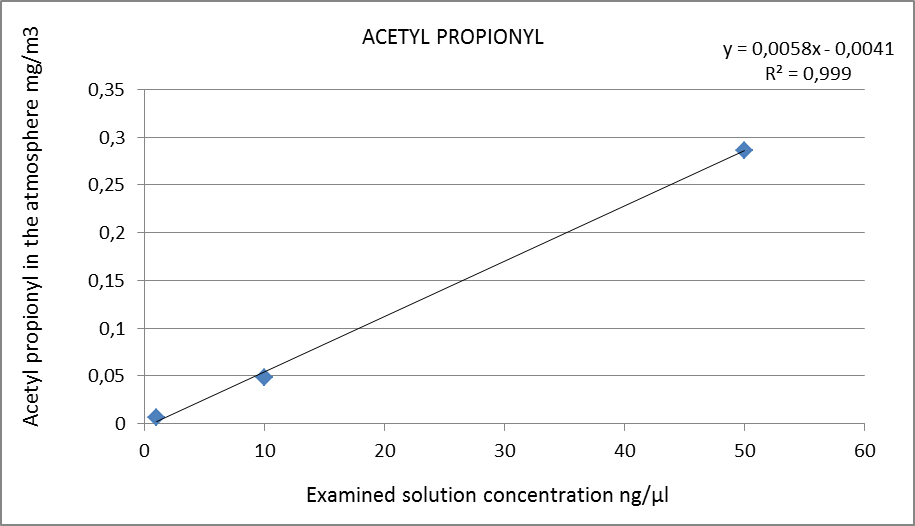


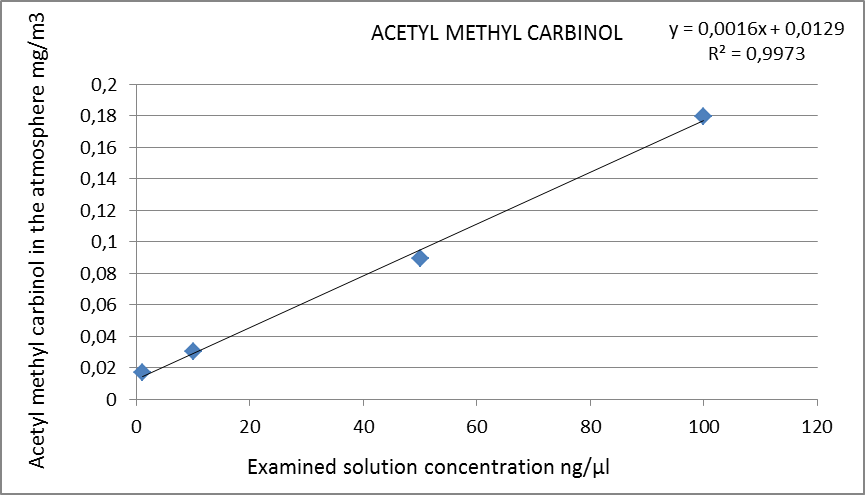


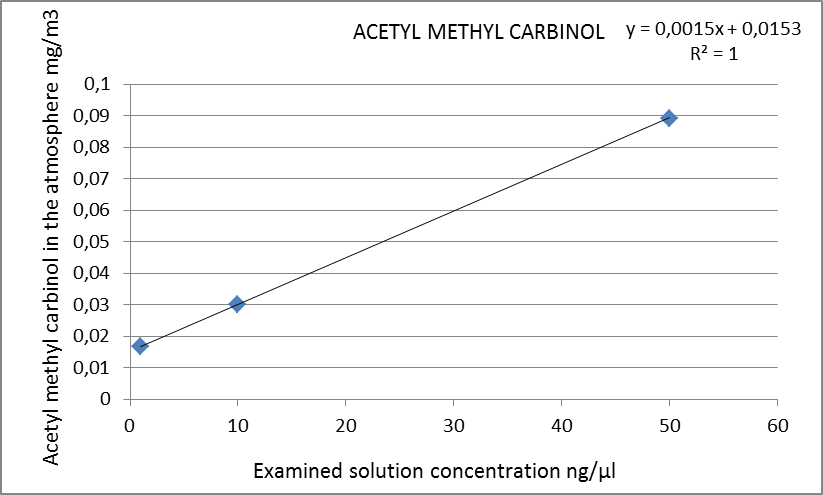


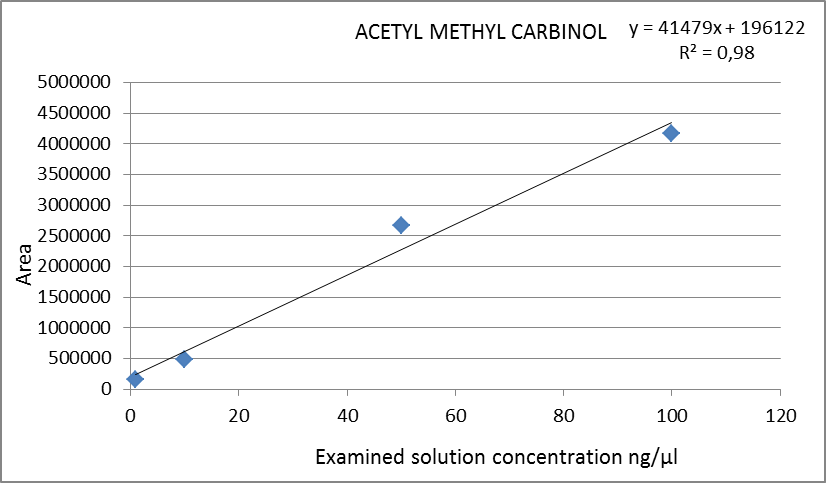


Conclusion : The target molecule concentration increases in a consistent manner depending

on the increase in the concentration of the solutions examined. The response is

linear for acetyl propionyl and for acetyl methyl carbinol with respective

correlation coefficients of 0.9887 and 0.9973. However, diacetyl (r2 = 0.9175)

linearity observed was not so good. Indeed, saturation of the sampling

devices was observed for the 100 ng/µl (2.8%) solution. If this last point is not

taken into account, the correlation coefficient rises to 0.9995.

Generally, the results of the tests carried out in a sealed enclosure (optimum

sampling conditions) are satisfactory and consistent with regard to the technique used.

1. ***SAMPLING TESTS IN A DEDICATED PLACE ON OUR***

***PRODUCTION PLANT***

**VIII-1 Study of the influence of the variation pumping time on the quantity of pollutants sampled (at set test flow rate and concentration)**

To study the influence of the pumping time on the quantity of target molecules sampled

in the atmosphere, we carried out several pumping tests in a dedicated place on our production

plant by varying only the pumping time; i.e. by maintaining the concentration of the solution

studied at the set level (5% of diacetyl, acetyl propionyl and acetyl methyl carbinol) as well

as the pumping flow rate (50 ml/minutes).

Results :

**POST “WEIGHING”: 3 pumps at 20 cm above the solution (Pumping time 5 MIN,**

**Flow : 50ML/MIN)**

**POST “WEIGHING”: 3 pumps at 20 cm above the solution (Pumping time 10 MIN,**

**Flow : 50ML/MIN)**

**POST “WEIGHING”: 3 pumps at 20 cm above the solution (Pumping time 20 MIN,**

**Flow : 50ML/MIN)**

**POST “WEIGHING”: 3 pumps at 20 cm above the solution (Pumping time 30 MIN,**

**Flow : 50ML/MIN)**


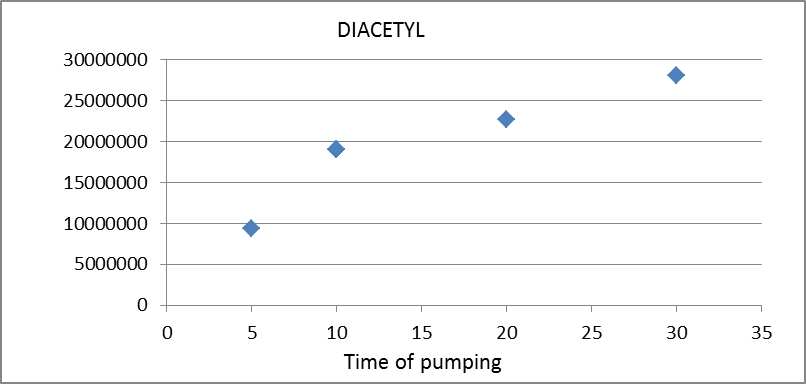


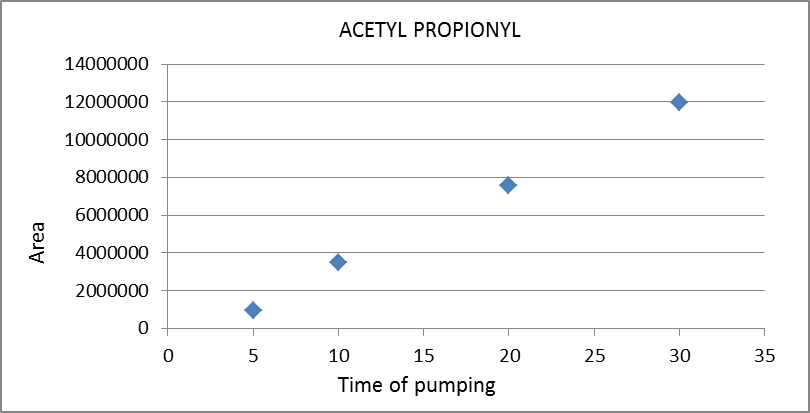


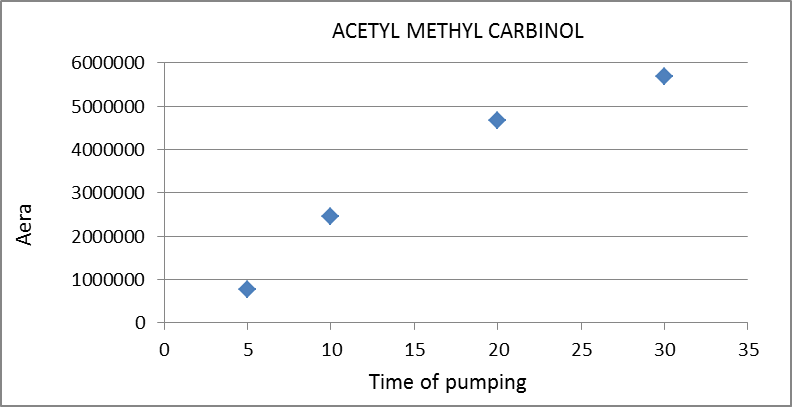


CONCLUSION :

When the pumping time at a constant flow rate is varied (thus variation in the volume

sampled) and the response obtained is measured, a logical proportionality between the quantity

of target molecules absorbed in the device and the volume of air sampled is observed.

The concentrations (in mg/m^3^) obtained for the different pumping times show that the results

are consistent (same order of magnitude), except in the case of Diacetyl for which we obtained

content values which collapse after 20 minutes of pumping. A breakthrough phenomenon cannot

be the only explanation since the tests consist of three tubes in series and significant quantities

of our target molecules are not found in the intermediate and final tubes. This test which was

carried out in a dedicated place on our production plant (closed room without ventilation)

and, consequently, in relatively favorable conditions, underlines the limits in terms of accuracy of

such a technique in the analysis of work atmospheres.

**VIII-2- Study of the influence of the concentration of the examined solution (at constant flow rate and sampling time).**

To study the influence of the concentration of the solutions examined in relation to the quantity

of target molecules sampled in the atmosphere, several pumping tests were carried out in a

dedicated place on our production plant by varying only the concentration of the solutions

examined (from 0.0048% to 1.25%), and by keeping both the pumping time and the pumping

flow rate (50 ml/minutes) constant.


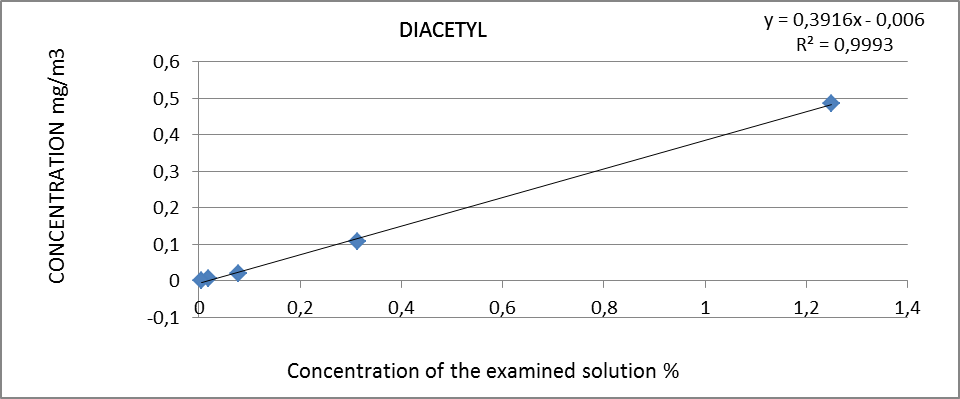


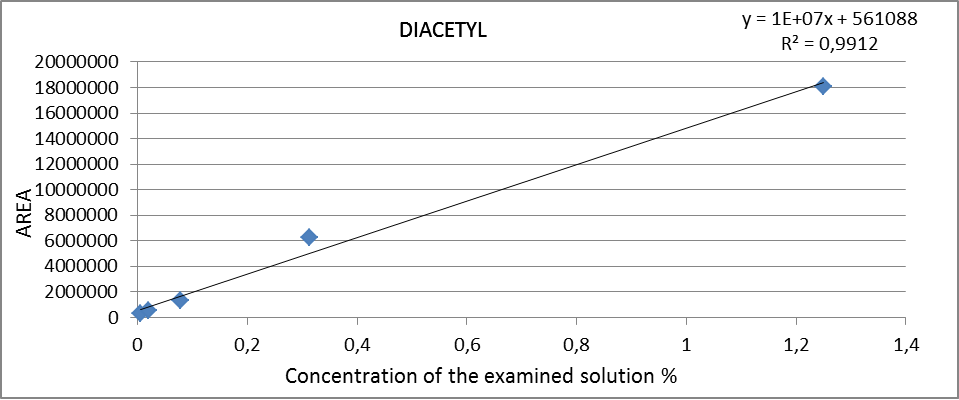


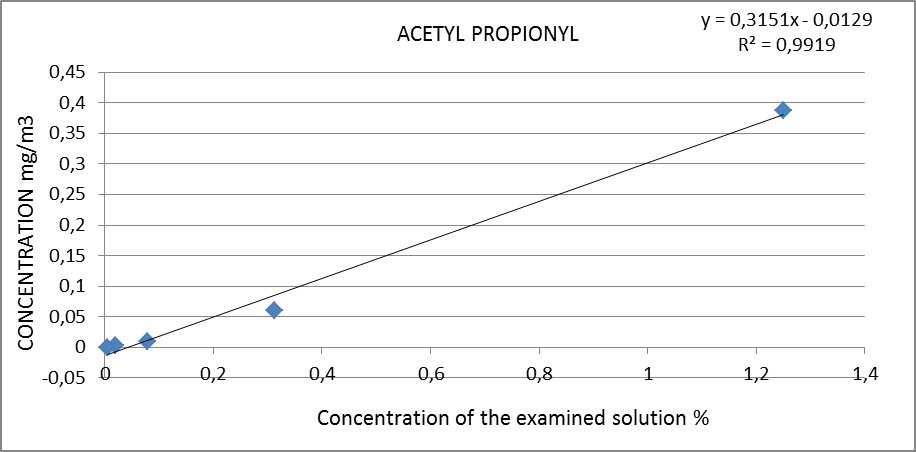


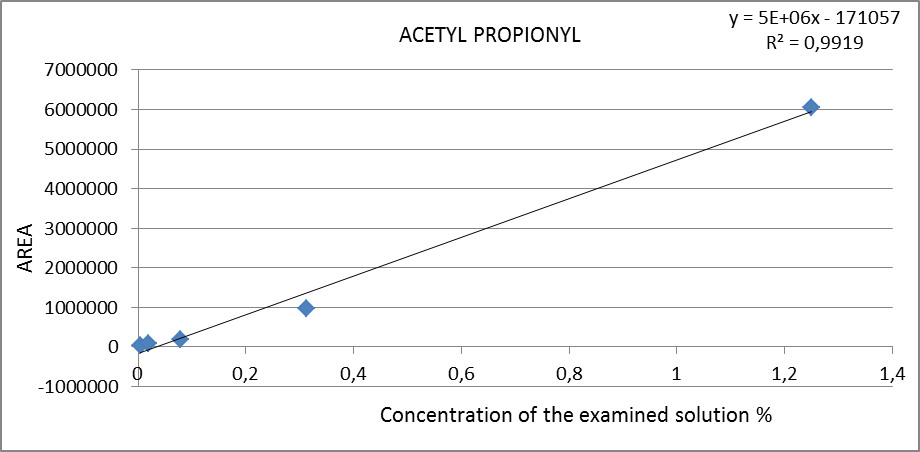


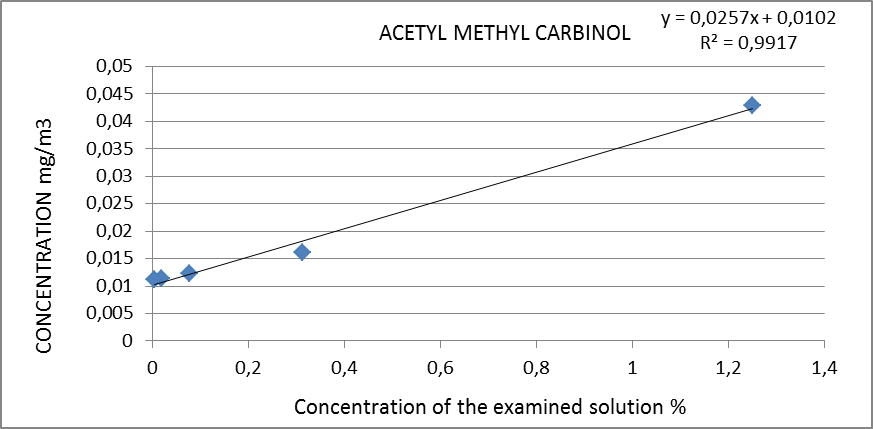


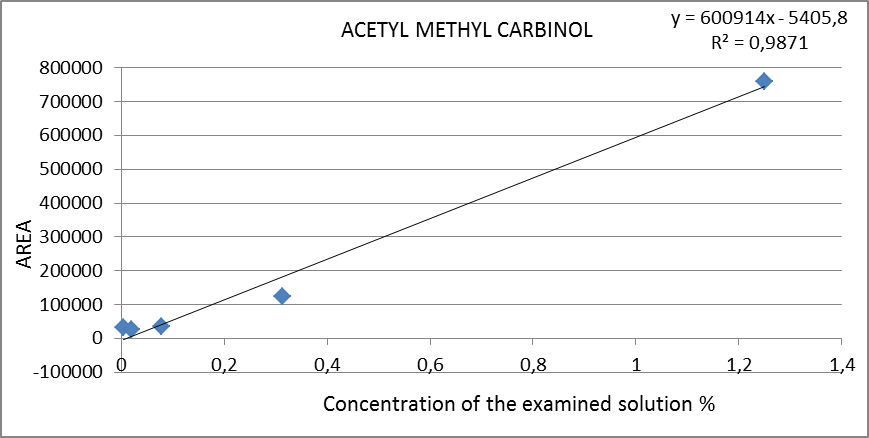


**Conclusion :** When similar volumes of air with increasing concentrations of 0.0048% to 1.25% are sampled, a consistent proportionality between the concentration of the solution examined and the acceptable response obtained (see linearities) is observed. Similar tests carried out previously in the laboratory in a sealed enclosure on a volume of 5 liters had also given linear results.

***General Conclusion***

The validation of the analysis method for Diacetyl, Acetyl propionyl and Acetyl methyl carbinol present in the work atmosphere, by entrapment on a Tenax medium and then thermal desorption, was carried out under optimal, controlled conditions (in terms of the pollutant concentration/work room volume ratio, positioning of the sampling devices, volume of atmosphere sampled, presence of co-pollutants, etc.). It proved to be repeatable, reproducible, linear (results obtained in the dedicated room on our production plant were not very satisfactory but should be weighted by an analytical technique greatly affected by the environmental parameters), with little interference from significant variations in the humidity level and small variations in temperature.

Insofar as significant delays will certainly occur between sampling in the subsidiaries and their analyses at BSL, we also carried out tests in order to estimate a maximum storage time for our sample tubes before analysis.

These tests revealed good storage for our samples up to 16 days before analysis. Moreover, we did not detect significant deterioration of our three target molecules over this period.

A leak test was successfully conducted in much stricter operating conditions than those recommended by the standard, NF IN 1076 (2*VL). Despite these extreme conditions, the devices proved to be impermeable in accordance with the criteria of this standard, thus guaranteeing the integrity of the samples and the absence of cross-pollution during the transport of these tubes.

The various tests conducted, either in the laboratory, or in the fire room at La Sarrée, enabled us to raise a certain number of problems directly and significantly affecting the samples:

- *The room volume/concentration of the examined solution ratio*: the volume of the room in which the sampling is carried out will make it possible to dilute the quantities of pollutants emitted into the air. Depending on the quantity and concentration of the solutions to be examined and the total volume of the room, the sampling devices will be saturated at a speed which is impossible to predict. Only a linearity study can show a saturation phenomenon. The tests carried out in the dedicated room (approximately 50 m^3^) helped to determine that in the sampling conditions validated in the laboratory (30 minutes at 50 ml/min), a solution of our target molecules of 1.25% in 100 ml of ethanol represented the concentration limit above which the tubes were saturated. The emission rate or quantity of emitted pollutants is difficult to estimate because it is related to the evaporation rate for each molecule, to temperature, to contact surface between the examined solution and the atmosphere, to the flow rate and type of ventilation, to the parameters of mixture of pollutants, etc.
- *The presence of “co-pollutants” i.e. of molecules not studied but present in large quantity in the atmosphere will also affect the analytical results on two levels:*
  1. On the sampling device by a competition phenomenon during adsorption stage: in fact all molecules present in the sampling atmosphere, other than those examined, will be in competition and will attach themselves to the sampling device depending on their affinity for the medium. If breakthrough volumes for the “undesired pollutants” are close to those for the molecules studied, it will then be impossible to eliminate them, thus invalidating the samples.
  2. At the analytical level, these “undesired pollutants” will also affect the results because, if they cannot be eliminated during the analytical stages of partial pre-desorption or pre-purges, their presence will generate overpressures and variations in split flows during the analysis by thermal desorption, thus affecting the repeatability and, consequently, the accuracy of the analyses. This impact will be just as great as the concentration of the “undesired pollutants” will be large.

*The weak point of this analytical technique lies in its lack of robustness due to a significant number of parameters likely to interfere, both during sampling and analysis:*

- Presence of unforeseeable co-pollutants: the parameters at the various sampling locations will be very different from each other and the field conditions cannot be anticipated. If the conditions encountered in the field are very different from those encountered for development, it will be necessary to ensure that the method remains applicable.
- The room volume/concentration of the examined solution ratio: depending on the atmospheric concentration of the target molecules and volume of the room, the sampling volume could prove to be too high (saturation of the sampling devices) or conversely too low to reach the detection and/or quantification threshold. No anticipation of the field parameters is possible.

- If the field temperature is very different from that encountered during the development of the method (20 to 25°C), the saturation of the tubes will be accelerated, the pumping time and/or flow rate could prove to be too great, thus accelerating the possible saturation of the sampling devices.
