# Supplementary material for: Respiratory Health – Exposure Measurements and Modeling in the Fragrance and Flavour Industry
Source: PLoS One. 2016 Feb 10;11(2):e0148769. doi: 10.1371/journal.pone.0148769 (PMC4749324; doi:10.1371/journal.pone.0148769)
Supplement: S1 Table — Chemical names and CAS numbers of the 27 substances selected for the reason that they are handled in large amounts and represent potentially higher risks. (DOCX) [file pone.0148769.s002.docx]

**S1 Table. List of volatile organic substances selected and analyzed in the present study.**

| **Name of the Molecule** | **CAS No.** | **Vapor pressure (mbar)** | **Analyzed substances** |
| --- | --- | --- | --- |
| 1. ACETALDEHYDE | 75-07-0 | 1007 | ■ |
| 1. ACETONE | 67-64-1 |  | □ |
| 1. ACETYL METHYL CARBINOL | 513-86-0 | 2,7 | ■ |
| 1. ACETYL PROPIONYL (2,3-PENTANEDIONE) | 600-14-6 | 32 | ■ |
| 1. AMMONIUM SULFIDE | 12124-99-1 |  | □ |
| 1. BENZALDEHYDE | 100-52-7 | 1,3 | ■ |
| 1. BENZENE | 71-43-2 | 100 | 🞑 |
| 1. BENZYL ALCOHOL | 100-51-6 | 0,132 | ■ |
| 1. BUTYL ACETATE | 123-86-4 | 11,6 | ■ |
| 1. BUTYL ALCOHOL | 71-36-3 | 5,8 | ■ |
| 1. BUTYLAMINE | 109-73-9 |  | □ |
| 1. CINNAMALDEHYDE | 104-55-2 | 141 | ■ |
| 1. CROTONALDEHYDE | 123-73-9 |  | □ |
| 1. CYCLOHEXANONE | 108-94-1 |  | □ |
| 1. DIACETYL | 431-03-8 | 57 | ■ |
| 1. DIMETHYL DISULFIDE | 624-92-0 |  | □ |
| 1. DIMETHYL SULFIDE | 75-18-3 | 532 | ■ |
| 1. ETHANOL | 64-17-5 | 59 | ■ |
| 1. ETHYL ACETATE | 141-78-6 | 76 | ■ |
| 1. ETHYL ACRYLATE | 140-88-5 |  | □ |
| 1. ETHYL FORMIATE | 109-94-4 | 256 | ■ |
| 1. ETHYL LACTATE | 97-64-3 | 2,2 | ■ |
| 1. FURFURAL | 98-01-1 | 2,26 | ■ |
| 1. FURFURYL ALCOHOL | 98-00-0 | 0,53 | 🞑 |
| 1. HEXANE | 110-54-3 | 160 | ■ |
| 1. ISOAMYL ACETATE | 123-92-2 | 5,3  4 | ■ |
| 1. ISOAMYL ALCOHOL | 123-51-3 | 4 | ■ |
| 1. ISOBUTYL ALCOHOL | 78-83-1 | 11,7 | ■ |
| 1. ISOBUTYRIC ACID | 79-31-2 |  | □ |
| 1. ISOBUTYRIC ALDEHYDE | 78-84-2 |  | □ |
| 1. ISOPHORONE | 78-59-1 |  | □ |
| 1. ISOPROPYL ALCOHOL | 67-63-0 | 44 | ■ |
| 1. ISOPROPYL MERCAPTAN | 75-33-2 |  | □ |
| 1. ISOVALERIC ALDEHYDE | 590-86-3 | 57,9 | 🞑 |
| 1. METHANOL | 67-56-1 | 123 | ■ |
| 1. METHYL ACETATE | 79-20-9 |  | □ |
| 1. METHYL ETHYL BUTYL KETONE | 541-85-5 | 3 | ■ |
| 1. METHYL MERCAPTAN | 74-93-1 |  | □ |
| 1. o-CRESOL | 95-48-7 |  | □ |
| 1. p-CRESOL | 106-44-5 |  | □ |
| 1. PHENOL | 108-95-2 |  | □ |
| 1. PRENOL (3-METHYL-2-BUTEN-1-OL) | 556-82-1 |  | □ |
| 1. PROPIONALDEHYDE | 123-38-6 |  | □ |
| 1. PROPYL ACETATE | 109-60-4 | 33 | ■ |
| 1. PROPYL ALCOHOL | 71-23-8 | 19,4 | ■ |
| 1. PROPYL MERCAPTAN | 107-03-9 |  | □ |
| 1. PYRIDINE | 110-86-1 |  | □ |
| 1. PYRROLIDINE | 123-75-1 |  | □ |
| 1. TRIMETHYLAMINE 45% (AQUEOUS) | 75-50-3 |  | □ |

■ *analyzed in the present study.*

🞑 *subjected to annual monitoring by an accredited laboratory.*

□ *selected for further investigation.*
