# Supplementary material for: Respiratory Health – Exposure Measurements and Modeling in the Fragrance and Flavour Industry
Source: PLoS One. 2016 Feb 10;11(2):e0148769. doi: 10.1371/journal.pone.0148769 (PMC4749324; doi:10.1371/journal.pone.0148769)
Supplement: S2 Table — Full detail of the nature of the absorbents, the pumping and desorption parameters, and analytical methodologies. (DOCX) [file pone.0148769.s003.docx]

**S2 Table. MANE sampling and analytical methods.**

| ***Molecule(s)*** | ***Adsorbent*** | ***Pump flow in mL/min*** | ***Pumping time in min*** | ***Desorption mode*** | ***Mass range, SIM/SCAN Mode*** | ***Column parameters***  ***Flow: 1.6 mL/min***  ***Velocity: 33 cm/s*** |
| --- | --- | --- | --- | --- | --- | --- |
| *Ethanol* | *Tenax1* | *15* | *<15* | *Thermal desorption at 200°C* | *m/z: 35 to 350*  *Ions: 45.,46* | *50°C for 10 min 20°C/min→240°C, 15min* |
| *Aldehydes with Number C<4* | *Impregnated silica2* | *40* | *<45* | *Liquid*  *Elution with methylene chloride* | *m/z: 35 to 350*  *Ions for acetaldehyde: 224* | *50°C for 10 min 20°C/min→240°C, 15 min* |
| *Diacetyl*  *Acetyl propionyl Acetyl methyl carbinol* | *Tenax* | *50* | *<45* | *Thermal desorption at 220°C* | *m/z: 35 to 200*  *Ions (respectively): 43.,86*  *57.,100*  *45,.88* | *70°C for 5 min 5°C/min→120°C*  *25°C/min→200°C* |
| *Dimethylsulfide* | *Tenax* | *15* | *<15* | *Thermal desorption at 200°C* | *m/z: 35 to 350*  *Ions: 62,47* | *60°C for 5 min 8°C/min→220°C* |
| *Other molecules: Ethyle acetate*  *Butyl acetate, etc...* | *Tenax* | *50* | *<45* | *Thermal desorption at 220°C* | *m/z: 35 to 350* | *50°C for 10 min 20°C/min→240°C, 15 min* |

*1Tenax*®*: 2,6 Diphenyl p phenylene oxide*

*2Silica: impregnated with pentafluorophenyl hydrazine*

*Thermal desorber UNITY MARKES International*

*ChromatoGas chromatography Agilent 6890N: Column AGILENT Innowax – 60 m x 320 μm x 0,25 μm*

*Quadripoloar mass spectrometer AGILENT TECHNOLOGIES 5973 Network*

*Calibration range: deposit of 1µL of each solution on a Tenax tube or impregnated silica (the concentration ranges are to be defined depending on the sample).*
