# Supplementary material for: Respiratory Health – Exposure Measurements and Modeling in the Fragrance and Flavour Industry
Source: PLoS One. 2016 Feb 10;11(2):e0148769. doi: 10.1371/journal.pone.0148769 (PMC4749324; doi:10.1371/journal.pone.0148769)
Supplement: S3 Table — The volatile compounds that were quantified in the present study were selected for their potentially high inhalation and respiratory risks, according to the risks phrases listed here. R-phrases and their corresponding symbols according to DSD: Dangerous Substances Directive (Directive 67/548/CEE); DPD: Dangerous Preparations Directive (Directive 1999/45/CE) and CLP Regulation (EC Regulation 1272/2008) are presented here in a table form. (DOCX) [file pone.0148769.s004.docx]

**S3 Table. Risk phrases.**

| Label elements  of the DSD/DPD* directives | Label elements  of the CLP** regulation |
| --- | --- |
|   R20 Harmful by inhalation |   H332 Harmful if inhaled |
|   R23 Toxic by inhalation |   H331 Toxic if inhaled |
|   R26 Very toxic by inhalation |   H330 Fatal if inhaled |
|   R37 Irritating to respiratory system |   H335 May cause respiratory irritation |
|   R42 May cause sensitisation by inhalation |   H334 May cause allergy or asthma symptoms or breathing difficulties if inhaled |
|   R48 Danger of serious damage to health by prolonged exposure |   H372 Causes damage to organs through prolonged or repeated exposure |
|   R49 May cause cancer by inhalation |   H350i May cause cancer by inhalation |
| R67 Vapors may cause drowsiness and dizziness |   H336 May cause drowsiness or dizziness |

*DSD: Dangerous Substances Directive (Directive 67/548/CEE); DPD: Dangerous Preparations Directive (Directive 1999/45/CE). Council Directive 67/548/EEC of 27 June 1967 on the approximation of laws, regulations and administrative provisions relating to the classification, packaging and labelling of dangerous substances. Directive 1999/45/EC of the European Parliament and of the Council of 31 May 1999 concerning the approximation of the laws, regulations and administrative provisions of the Member States relating to the classification, packaging and labelling of dangerous preparations.

**Regulation (EC) No 1272/2008 on classification, labelling and packaging of substances and mixtures (the CLP Regulation or simply “CLP”) which entered into force on 20 January 2009 in the EU countries and has now relevance for European Economic Area (EEA) countries (i.e. it is implemented in the EU countries and in Norway, Iceland and Liechtenstein).
