# Supplementary material for: Respiratory Health – Exposure Measurements and Modeling in the Fragrance and Flavour Industry
Source: PLoS One. 2016 Feb 10;11(2):e0148769. doi: 10.1371/journal.pone.0148769 (PMC4749324; doi:10.1371/journal.pone.0148769)
Supplement: S4 Table — Comprehensive sample results collected in V. MANE FILS workshops. (DOCX) [file pone.0148769.s005.docx]

**S4 Table. Full data table.**

| **Substance** | **Type of operation** | **Handled quantity (kg)** | **Measurement carried out on an operator (ppm)** | **MANE model forecast (ppm)** | **ECETOC model forecast (ppm)** |
| --- | --- | --- | --- | --- | --- |
| ACETALDEHYDE | Transfering | 240 | 85 | 106 | 2,7 |
| ACETALDEHYDE | Transfering | 270 | 47 | 106 | 2,7 |
| ACETALDEHYDE | Transfering | 255 | 37 | 76 | 0,9 |
| ACETALDEHYDE | Transfering | 270 | 77 | 76 | 0,9 |
| ACETALDEHYDE | Transfering | 191 | 83 | 106 | 2,7 |
| ACETYL METHYL CARBINOL | Weighing | 0,25 | 0,58 | 2,7 | 0,30 |
| ACETYL METHYL CARBINOL | Weighing | 0,25 | 0,54 | 2,7 | 0,30 |
| ACETYL METHYL CARBINOL | Weighing | 0,25 | 0,02 | 2,7 | 0,30 |
| ACETYL METHYL CARBINOL | Weighing | 1 | 2,3 | 2,7 | 0,30 |
| ACETYL METHYL CARBINOL | Weighing | 1 | 1,8 | 2,7 | 0,30 |
| ACETYL METHYL CARBINOL | Weighing | 1 | 0,54 | 2,7 | 0,30 |
| ACETYL METHYL CARBINOL | Weighing | 2,5 | 0,55 | 2,7 | 0,30 |
| ACETYL METHYL CARBINOL | Weighing | 2,5 | 0,44 | 2,7 | 0,30 |
| ACETYL METHYL CARBINOL | Weighing | 2,5 | 0,43 | 2,7 | 0,30 |
| ACETYL METHYL CARBINOL | Packaging | 1 | 0,12 | 0,21 | 0,03 |
| ACETYL METHYL CARBINOL | Packaging | 0,25 | 0,02 | 0,05 | 0,02 |
| ACETYL METHYL CARBINOL | Packaging | 0,25 | 0,01 | 0,05 | 0,02 |
| ACETYL METHYL CARBINOL | Packaging | 0,25 | 0,04 | 0,27 | 0,03 |
| ACETYL METHYL CARBINOL | Packaging | 2,5 | 0,03 | 0,42 | 0,09 |
| ACETYL METHYL CARBINOL | Packaging | 2,5 | 0,02 | 0,42 | 0,09 |
| ACETYL PROPIONYL | Weighing | 1 | 0,50 | 1,7 | 0,15 |
| ACETYL PROPIONYL | Weighing | 0,5 | 0,26 | 1,7 | 0,15 |
| ACETYL PROPIONYL | Weighing | 2,5 | 0,26 | 1,7 | 0,15 |
| ACETYL PROPIONYL | Weighing | 0,5 | 0,22 | 1,7 | 0,15 |
| ACETYL PROPIONYL | Weighing | 1 | 0,15 | 1,7 | 0,15 |
| ACETYL PROPIONYL | Weighing | 2,5 | 0,13 | 1,7 | 0,15 |
| BENZALDEHYDE | Weighing | 161 | 0,01 | 7,9 | 1,0 |
| BENZALDEHYDE | Packaging | 161 | 0,05 | 4,4 | 0,60 |
| BENZALDEHYDE | Weighing | 56 | 0,03 | 0,47 | 0,03 |
| BENZALDEHYDE | Weighing | 0,32 | 0,01 | 0,47 | 0,02 |
| BENZALDEHYDE | Packaging | 0,32 | 0,01 | 0,47 | 0,02 |
| BENZALDEHYDE | Weighing | 0,4 | 0,04 | 0,47 | 0,09 |
| BENZALDEHYDE | Weighing | 0,25 | 0,11 | 0,47 | 0,03 |
| BENZALDEHYDE | Weighing | 0,25 | 0,06 | 0,47 | 0,03 |
| BENZALDEHYDE | Weighing | 0,25 | 0,04 | 0,47 | 0,03 |
| BENZALDEHYDE | Weighing | 1 | 0,03 | 0,47 | 0,03 |
| BENZALDEHYDE | Weighing | 1 | 0,03 | 0,47 | 0,03 |
| BENZALDEHYDE | Weighing | 1 | 0,002 | 0,47 | 0,03 |
| BENZALDEHYDE | Weighing | 2,5 | 0,04 | 0,47 | 0,03 |
| BENZALDEHYDE | Weighing | 2,5 | 0,03 | 0,47 | 0,03 |
| BENZALDEHYDE | Weighing | 2,5 | 0,02 | 0,47 | 0,03 |
| BENZALDEHYDE | Packaging | 1 | 0,88 | 0,95 | 0,003 |
| BENZALDEHYDE | Packaging | 0,25 | 0,14 | 0,24 | 0,002 |
| BENZALDEHYDE | Packaging | 2,5 | 0,95 | 1,9 | 0,01 |
| BENZALDEHYDE | Packaging | 0,25 | 0,10 | 0,24 | 0,002 |
| BENZALDEHYDE | Packaging | 0,25 | 0,09 | 0,24 | 0,003 |
| BENZALDEHYDE | Packaging | 1 | 0,32 | 0,95 | 0,003 |
| BENZALDEHYDE | Packaging | 1 | 0,20 | 0,95 | 0,003 |
| BENZALDEHYDE | Packaging | 2,5 | 0,08 | 1,9 | 0,01 |
| BENZALDEHYDE | Packaging | 2,5 | 0,07 | 1,9 | 0,01 |
| BENZENE | Weighing | 0,001 | 0,01 | 2,3 | 0,9 |
| BENZENE | Weighing | 0,001 | 0,08 | 2,3 | 0,9 |
| BENZENE | Weighing | 0,001 | 0,09 | 0,45 | 0,9 |
| BENZENE | Weighing | 0,001 | 0,09 | 0,45 | 0,9 |
| BENZENE | Weighing | 0,001 | 0,05 | 2,3 | 0,9 |
| BENZENE | Weighing | 0,001 | 0,29 | 0,45 | 0,9 |
| BENZENE | Weighing | 0,001 | 0,25 | 0,45 | 0,9 |
| BENZENE | Weighing | 0,001 | 0,21 | 0,45 | 0,9 |
| BENZENE | Weighing | 0,001 | 0,25 | 0,45 | 0,9 |
| BENZENE | Weighing | 0,001 | 0,19 | 0,45 | 0,9 |
| BENZENE | Weighing | 0,001 | 0,19 | 0,45 | 0,9 |
| BENZENE | Weighing | 0,001 | 0,13 | 0,45 | 0,9 |
| BENZENE | Weighing | 0,001 | 0,12 | 0,45 | 0,9 |
| BENZENE | Weighing | 0,001 | 0,11 | 0,45 | 0,9 |
| BENZENE | Weighing | 0,001 | 0,17 | 0,45 | 0,9 |
| BENZENE | Weighing | 0,001 | 0,15 | 0,45 | 0,9 |
| BENZENE | Weighing | 0,001 | 0,15 | 0,45 | 0,9 |
| BENZENE | Weighing | 0,001 | 0,20 | 0,45 | 0,9 |
| BENZENE | Weighing | 0,001 | 0,17 | 0,45 | 0,9 |
| BENZENE | Weighing | 0,001 | 0,15 | 0,45 | 0,9 |
| BENZYL ALCOHOL | Weighing | 0,25 | 0,01 | 0,06 | 0,03 |
| BENZYL ALCOHOL | Weighing | 0,25 | 0,002 | 0,06 | 0,03 |
| BENZYL ALCOHOL | Weighing | 0,25 | 0,001 | 0,06 | 0,03 |
| BENZYL ALCOHOL | Weighing | 1 | 0,01 | 0,06 | 0,03 |
| BENZYL ALCOHOL | Weighing | 1 | 0,001 | 0,06 | 0,03 |
| BENZYL ALCOHOL | Weighing | 2,5 | 0,004 | 0,06 | 0,03 |
| BENZYL ALCOHOL | Weighing | 2,5 | 0,003 | 0,06 | 0,03 |
| BENZYL ALCOHOL | Weighing | 2,5 | 0,002 | 0,06 | 0,03 |
| BENZYL ALCOHOL | Packaging | 0,25 | 0,02 | 0,03 | 0,002 |
| BENZYL ALCOHOL | Packaging | 0,25 | 0,02 | 0,03 | 0,002 |
| BENZYL ALCOHOL | Packaging | 1 | 0,07 | 0,12 | 0,003 |
| BENZYL ALCOHOL | Packaging | 1 | 0,06 | 0,12 | 0,003 |
| BENZYL ALCOHOL | Packaging | 2,5 | 0,06 | 0,12 | 0,01 |
| BENZYL ALCOHOL | Packaging | 1 | 0,05 | 0,12 | 0,003 |
| BENZYL ALCOHOL | Packaging | 2,5 | 0,04 | 0,12 | 0,01 |
| BENZYL ALCOHOL | Packaging | 0,25 | 0,01 | 0,03 | 0,00 |
| BENZYL ALCOHOL | Packaging | 2,5 | 0,02 | 0,12 | 0,01 |
| BENZYL ALCOHOL | Weighing | 0,15 | 0,06 | 0,06 | 0,015 |
| BENZYL ALCOHOL | Weighing | 3 | 0,06 | 0,06 | 0,015 |
| BENZYL ALCOHOL | Weighing | 3 | 0,06 | 0,06 | 0,015 |
| BENZYL ALCOHOL | Weighing | 0,015 | 0,04 | 0,06 | 0,015 |
| BUTYL ACETATE | Weighing | 0,137 | 0,18 | 0,89 | 0,15 |
| BUTYL ACETATE | Weighing | 0,15 | 0,02 | 0,89 | 0,15 |
| BUTYL ACETATE | Weighing | 1 | 0,12 | 0,89 | 0,15 |
| BUTYL ACETATE | Weighing | 1 | 0,08 | 0,89 | 0,15 |
| BUTYL ACETATE | Weighing | 1 | 0,01 | 0,89 | 0,15 |
| BUTYL ALCOHOL | Weighing | 0,25 | 0,13 | 0,66 | 0,30 |
| BUTYL ALCOHOL | Weighing | 0,25 | 0,05 | 0,66 | 0,30 |
| BUTYL ALCOHOL | Weighing | 0,25 | 0,03 | 0,66 | 0,30 |
| BUTYL ALCOHOL | Weighing | 1 | 0,05 | 0,66 | 0,30 |
| BUTYL ALCOHOL | Weighing | 1 | 0,05 | 0,66 | 0,30 |
| BUTYL ALCOHOL | Weighing | 1 | 0,02 | 0,66 | 0,30 |
| BUTYL ALCOHOL | Weighing | 2,5 | 0,08 | 0,66 | 0,30 |
| BUTYL ALCOHOL | Weighing | 2,5 | 0,05 | 0,66 | 0,30 |
| BUTYL ALCOHOL | Weighing | 2,5 | 0,04 | 0,66 | 0,30 |
| BUTYL ALCOHOL | Packaging | 2,5 | 2,1 | 2,6 | 0,09 |
| BUTYL ALCOHOL | Packaging | 0,25 | 0,10 | 0,33 | 0,02 |
| BUTYL ALCOHOL | Packaging | 1 | 0,31 | 1,3 | 0,03 |
| BUTYL ALCOHOL | Packaging | 0,25 | 0,08 | 0,33 | 0,02 |
| BUTYL ALCOHOL | Packaging | 0,25 | 0,06 | 0,33 | 0,03 |
| BUTYL ALCOHOL | Packaging | 1 | 0,17 | 1,3 | 0,03 |
| BUTYL ALCOHOL | Packaging | 2,5 | 0,13 | 1,3 | 0,09 |
| BUTYL ALCOHOL | Packaging | 2,5 | 0,09 | 1,3 | 0,09 |
| BUTYL ALCOHOL | Weighing | 0,15 | 0,64 | 0,66 | 0,15 |
| BUTYL ALCOHOL | Weighing | 0,15 | 0,63 | 0,66 | 0,15 |
| BUTYL ALCOHOL | Weighing | 0,03 | 0,57 | 0,66 | 0,15 |
| CINNAMIC ALDEHYDE | Weighing | 0,25 | 0,02 | 5,0 | 0,03 |
| CINNAMIC ALDEHYDE | Weighing | 0,25 | 0,02 | 5,0 | 0,03 |
| CINNAMIC ALDEHYDE | Weighing | 0,25 | 0,001 | 5,0 | 0,03 |
| CINNAMIC ALDEHYDE | Weighing | 1 | 0,002 | 5,0 | 0,03 |
| CINNAMIC ALDEHYDE | Weighing | 1 | 0,002 | 5,0 | 0,03 |
| CINNAMIC ALDEHYDE | Weighing | 2,5 | 0,01 | 5,0 | 0,03 |
| CINNAMIC ALDEHYDE | Weighing | 2,5 | 0,002 | 5,0 | 0,03 |
| CINNAMIC ALDEHYDE | Weighing | 2,5 | 0,001 | 5,0 | 0,03 |
| CINNAMIC ALDEHYDE | Packaging | 0,25 | 0,07 | 2,5 | 0,002 |
| CINNAMIC ALDEHYDE | Packaging | 0,25 | 0,07 | 2,5 | 0,002 |
| CINNAMIC ALDEHYDE | Packaging | 1 | 0,16 | 10 | 0,003 |
| CINNAMIC ALDEHYDE | Packaging | 1 | 0,14 | 10 | 0,003 |
| CINNAMIC ALDEHYDE | Packaging | 2,5 | 0,12 | 10 | 0,01 |
| CINNAMIC ALDEHYDE | Packaging | 2,5 | 0,10 | 10 | 0,01 |
| CINNAMIC ALDEHYDE | Packaging | 1 | 0,10 | 10 | 0,00 |
| CINNAMIC ALDEHYDE | Packaging | 2,5 | 0,06 | 10 | 0,01 |
| CINNAMIC ALDEHYDE | Packaging | 0,25 | 0,01 | 2,5 | 0,003 |
| DIACETYL | Transfering | 130 | 7,0 | 19 | 0,90 |
| DIACETYL | Transfering | 100 | 8,4 | 14 | 10,0 |
| DIACETYL | Transfering | 100 | 3,3 | 14 | 0,30 |
| DIACETYL | Weighing | 33 | 5,6 | 9,2 | 0,90 |
| DIACETYL | Weighing | 21 | 2,1 | 2,7 | 0,90 |
| DIACETYL | Packaging | 33 | 9,0 | 11 | 0,54 |
| DIACETYL | Packaging | 21 | 0,08 | 11 | 0,18 |
| DIACETYL | Transfering | 380 | 0,80 | 9,2 | 5,0 |
| DIACETYL | Spray drying | 380 | 0,05 | 2,7 | 0,15 |
| DIACETYL | Granulation | 18 | 0,27 | 2,7 | 0,15 |
| DIACETYL | Weighing | 0,25 | 0,58 | 2,7 | 0,30 |
| DIACETYL | Weighing | 0,25 | 0,44 | 2,7 | 0,30 |
| DIACETYL | Weighing | 0,25 | 0,28 | 2,7 | 0,30 |
| DIACETYL | Weighing | 1 | 0,68 | 2,7 | 0,30 |
| DIACETYL | Weighing | 1 | 0,52 | 2,7 | 0,30 |
| DIACETYL | Weighing | 1 | 0,35 | 2,7 | 0,30 |
| DIACETYL | Weighing | 2,5 | 0,85 | 2,7 | 0,30 |
| DIACETYL | Weighing | 2,5 | 0,52 | 2,7 | 0,30 |
| DIACETYL | Weighing | 2,5 | 0,49 | 2,7 | 0,30 |
| DIACETYL | Packaging | 0,25 | 0,65 | 1,4 | 0,02 |
| DIACETYL | Packaging | 1 | 2,5 | 5,5 | 0,03 |
| DIACETYL | Packaging | 1 | 2,2 | 5,5 | 0,03 |
| DIACETYL | Packaging | 1 | 2,0 | 5,5 | 0,03 |
| DIACETYL | Packaging | 2,5 | 1,7 | 5,5 | 0,09 |
| DIACETYL | Packaging | 2,5 | 1,4 | 5,5 | 0,09 |
| DIACETYL | Packaging | 0,25 | 0,31 | 1,4 | 0,02 |
| DIACETYL | Packaging | 0,25 | 0,31 | 1,4 | 0,03 |
| DIACETYL | Weighing | 0,5 | 0,23 | 2,7 | 0,15 |
| DIACETYL | Weighing | 1 | 0,22 | 2,7 | 0,15 |
| DIACETYL | Weighing | 1 | 0,19 | 2,7 | 0,30 |
| DIACETYL | Weighing | 0,5 | 0,16 | 2,7 | 0,30 |
| DIMETHYL SULFIDE | Weighing | 1,2 | 0,8 | 9,6 | 0,45 |
| DIMETHYL SULFIDE | Weighing | 0,25 | 9,5 | 9,6 | 0,9 |
| DIMETHYL SULFIDE | Weighing | 0,25 | 9 | 9,6 | 0,90 |
| DIMETHYL SULFIDE | Weighing | 0,25 | 6 | 9,6 | 0,90 |
| DIMETHYL SULFIDE | Weighing | 1 | 6 | 9,6 | 0,90 |
| DIMETHYL SULFIDE | Weighing | 1 | 4 | 9,6 | 0,90 |
| DIMETHYL SULFIDE | Weighing | 1 | 0,20 | 9,6 | 0,90 |
| DIMETHYL SULFIDE | Weighing | 2,5 | 4 | 9,6 | 0,90 |
| DIMETHYL SULFIDE | Weighing | 2,5 | 3 | 9,6 | 0,90 |
| DIMETHYL SULFIDE | Weighing | 2,5 | 0,69 | 9,6 | 0,90 |
| DIMETHYL SULFIDE | Packaging | 1 | 19 | 19 | 0,09 |
| DIMETHYL SULFIDE | Packaging | 0,25 | 3 | 4,8 | 0,09 |
| DIMETHYL SULFIDE | Weighing | 1 | 0,72 | 32 | 1,5 |
| DIMETHYL SULFIDE | Weighing | 1 | 0,53 | 32 | 1,5 |
| DIMETHYL SULFIDE | Weighing | 1 | 0,33 | 32 | 1,5 |
| DIMETHYL SULFIDE | Weighing | 0,25 | 17 | 32 | 1,5 |
| DIMETHYL SULFIDE | Weighing | 0,25 | 16 | 32 | 1,5 |
| DIMETHYL SULFIDE | Weighing | 0,005 | 11 | 32 | 1,5 |
| DIMETHYL SULFIDE | Weighing | 0,005 | 8 | 32 | 1,5 |
| DIMETHYL SULFIDE | Weighing | 0,05 | 7 | 32 | 1,5 |
| DIMETHYL SULFIDE | Weighing | 0,05 | 3 | 32 | 1,5 |
| DIMETHYL SULFIDE | Weighing | 0,025 | 0,22 | 9,6 | 0,05 |
| DIMETHYL SULFIDE | Weighing | 0,05 | 8 | 9,6 | 0,05 |
| DIMETHYL SULFIDE | Weighing | 0,05 | 4 | 9,6 | 0,05 |
| DIMETHYL SULFIDE | Weighing | 0,25 | 14 | 32 | 1,5 |
| DIMETHYL SULFIDE | Weighing | 0,25 | 8 | 9,6 | 0,05 |
| DIMETHYL SULFIDE | Weighing | 0,5 | 0,44 | 9,6 | 0,05 |
| ETHANOL | Weighing | 98 | 136 | 140 | 0,3 |
| ETHANOL | Weighing | 3344 | 51 | 140 | 5 |
| ETHANOL | Packaging | 3344 | 91 | 140 | 10 |
| ETHANOL | Packaging | 334 | 21 | 42 | 0,3 |
| ETHANOL | Filtration | 3344 | 100 | 140 | 10 |
| ETHANOL | Packaging | 3344 | 35 | 43 | 0,3 |
| ETHANOL | Weighing | 3344 | 15 | 42 | 0,3 |
| ETHANOL | Weighing | 417 | 108 | 140 | 0,2 |
| ETHANOL | Weighing | 3344 | 26 | 140 | 10 |
| ETHANOL | Filtration | 3344 | 104 | 140 | 10 |
| ETHANOL | Filtration | 3344 | 97 | 140 | 10 |
| ETHANOL | Filtration | 3344 | 65 | 140 | 10 |
| ETHANOL | Packaging | 3344 | 27 | 43 | 0,3 |
| ETHANOL | Packaging | 3344 | 19 | 43 | 0,3 |
| ETHANOL | Packaging | 3344 | 18 | 43 | 0,3 |
| ETHANOL | Weighing | 46 | 17 | 42 | 0,2 |
| ETHANOL | Weighing | 46 | 10 | 42 | 0,2 |
| ETHANOL | Weighing | 46 | 9 | 42 | 0,2 |
| ETHANOL | Weighing | 32 | 23 | 24 | 0,2 |
| ETHANOL | Weighing | 32 | 19 | 24 | 0,2 |
| ETHANOL | Weighing | 5 | 17 | 24 | 0,2 |
| ETHANOL | Weighing | 5 | 7 | 24 | 0,2 |
| ETHANOL | Weighing | 5 | 5 | 24 | 0,2 |
| ETHANOL | Packaging | 137 | 25 | 42 | 0,2 |
| ETHANOL | Packaging | 15 | 13 | 34 | 0,1 |
| ETHANOL | Packaging | 96 | 12 | 42 | 0,2 |
| ETHYL ACETATE | Weighing | 0,662 | 2,9 | 3,5 | 0,3 |
| ETHYL ACETATE | Weighing | 0,02 | 2,3 | 3,5 | 0,3 |
| ETHYL ACETATE | Weighing | 240 | 0,54 | 12 | 5 |
| ETHYL ACETATE | Packaging | 240 | 4,4 | 33 | 6 |
| ETHYL ACETATE | Weighing | 0,67 | 0,2 | 3,5 | 0,9 |
| ETHYL ACETATE | Weighing | 0,92 | 0,84 | 3,5 | 0,9 |
| ETHYL ACETATE | Packaging | 23,6 | 3,9 | 47 | 6 |
| ETHYL ACETATE | Weighing | 0,25 | 0,34 | 3,5 | 0,3 |
| ETHYL ACETATE | Weighing | 0,25 | 0,19 | 3,5 | 0,3 |
| ETHYL ACETATE | Weighing | 0,25 | 0,18 | 3,5 | 0,3 |
| ETHYL ACETATE | Weighing | 1 | 1,3 | 3,5 | 0,3 |
| ETHYL ACETATE | Weighing | 1 | 0,71 | 3,5 | 0,3 |
| ETHYL ACETATE | Weighing | 1 | 0,05 | 3,5 | 0,15 |
| ETHYL ACETATE | Weighing | 2,5 | 0,82 | 3,5 | 0,3 |
| ETHYL ACETATE | Weighing | 2,5 | 0,70 | 3,5 | 0,3 |
| ETHYL ACETATE | Weighing | 2,5 | 0,66 | 3,5 | 0,3 |
| ETHYL ACETATE | Packaging | 2,5 | 11 | 14 | 0,09 |
| ETHYL ACETATE | Packaging | 0,25 | 1,1 | 1,8 | 0,015 |
| ETHYL ACETATE | Packaging | 1 | 3,7 | 7,0 | 0,03 |
| ETHYL ACETATE | Packaging | 1 | 3,6 | 7,0 | 0,03 |
| ETHYL ACETATE | Packaging | 1 | 3,3 | 7,0 | 0,03 |
| ETHYL ACETATE | Packaging | 2,5 | 2,6 | 7,0 | 0,09 |
| ETHYL ACETATE | Packaging | 2,5 | 2,3 | 7,0 | 0,09 |
| ETHYL ACETATE | Packaging | 0,25 | 0,48 | 1,8 | 0,03 |
| ETHYL ACETATE | Packaging | 0,25 | 0,46 | 1,8 | 0,015 |
| ETHYL FORMIATE | Weighing | 0,02 | 0,31 | 6,3 | 0,45 |
| ETHYL FORMIATE | Weighing | 0,18 | 0,62 | 6,3 | 0,45 |
| ETHYL FORMIATE | Packaging | 4 | 3,4 | 42 | 6,0 |
| ETHYL FORMIATE | Weighing | 0,25 | 0,78 | 6,3 | 0,90 |
| ETHYL FORMIATE | Weighing | 0,25 | 0,34 | 6,3 | 0,90 |
| ETHYL FORMIATE | Weighing | 0,25 | 0,33 | 6,3 | 0,90 |
| ETHYL FORMIATE | Weighing | 1 | 2,8 | 6,3 | 0,90 |
| ETHYL FORMIATE | Weighing | 1 | 1,1 | 6,3 | 0,90 |
| ETHYL FORMIATE | Weighing | 1 | 0,92 | 6,3 | 0,90 |
| ETHYL FORMIATE | Weighing | 2,5 | 2,2 | 6,3 | 0,90 |
| ETHYL FORMIATE | Weighing | 2,5 | 0,38 | 6,3 | 0,90 |
| ETHYL FORMIATE | Weighing | 2,5 | 0,33 | 6,3 | 0,90 |
| ETHYL FORMIATE | Packaging | 1 | 14 | 13 | 0,09 |
| ETHYL FORMIATE | Packaging | 2,5 | 8,8 | 13 | 0,27 |
| ETHYL FORMIATE | Packaging | 2,5 | 7,4 | 13 | 0,27 |
| ETHYL FORMIATE | Packaging | 1 | 6,9 | 13 | 0,09 |
| ETHYL FORMIATE | Packaging | 2,5 | 5,1 | 13 | 0,27 |
| ETHYL FORMIATE | Packaging | 0,25 | 1,2 | 3,2 | 0,05 |
| ETHYL FORMIATE | Packaging | 0,25 | 0,23 | 3,2 | 0,05 |
| ETHYL FORMIATE | Packaging | 1 | 0,87 | 13 | 0,09 |
| ETHYL FORMIATE | Packaging | 0,25 | 0,09 | 3,2 | 0,09 |
| ETHYL LACTATE | Weighing | 1 | 0,23 | 0,51 | 0,15 |
| ETHYL LACTATE | Weighing | 1 | 0,02 | 0,51 | 0,15 |
| ETHYL LACTATE | Weighing | 1 | 0,02 | 0,51 | 0,15 |
| ETHYL METHYL BUTYL KETONE | Weighing | 1 | 0,32 | 0,54 | 0,15 |
| ETHYL METHYL BUTYL KETONE | Weighing | 1 | 0,02 | 0,54 | 0,15 |
| ETHYL METHYL BUTYL KETONE | Weighing | 1 | 0,02 | 0,54 | 0,15 |
| FURFURAL | Weighing | 0,25 | 0,01 | 0,51 | 0,03 |
| FURFURAL | Weighing | 0,25 | 0,004 | 0,51 | 0,03 |
| FURFURAL | Weighing | 0,25 | 0,003 | 0,51 | 0,03 |
| FURFURAL | Weighing | 1 | 0,02 | 0,51 | 0,03 |
| FURFURAL | Weighing | 1 | 0,01 | 0,51 | 0,03 |
| FURFURAL | Weighing | 1 | 0,001 | 0,51 | 0,03 |
| FURFURAL | Weighing | 2,5 | 0,04 | 0,51 | 0,03 |
| FURFURAL | Weighing | 2,5 | 0,01 | 0,51 | 0,03 |
| FURFURAL | Weighing | 2,5 | 0,01 | 0,51 | 0,03 |
| FURFURAL | Packaging | 1 | 0,68 | 1,0 | 0,003 |
| FURFURAL | Packaging | 2,5 | 1,4 | 2,0 | 0,01 |
| FURFURAL | Packaging | 0,25 | 0,12 | 0,26 | 0,002 |
| FURFURAL | Packaging | 1 | 0,47 | 1,0 | 0,003 |
| FURFURAL | Packaging | 0,25 | 0,11 | 0,26 | 0,003 |
| FURFURAL | Packaging | 0,25 | 0,09 | 0,26 | 0,002 |
| FURFURAL | Packaging | 2,5 | 0,11 | 1,0 | 0,01 |
| FURFURAL | Packaging | 2,5 | 0,10 | 1,0 | 0,01 |
| FURFURYL ALCOHOL | Weighing | 0,25 | 0,02 | 0,24 | 0,03 |
| FURFURYL ALCOHOL | Weighing | 0,25 | 0,02 | 0,24 | 0,03 |
| FURFURYL ALCOHOL | Weighing | 0,25 | 0,01 | 0,24 | 0,03 |
| FURFURYL ALCOHOL | Weighing | 1 | 0,01 | 0,24 | 0,03 |
| FURFURYL ALCOHOL | Weighing | 1 | 0,01 | 0,24 | 0,03 |
| FURFURYL ALCOHOL | Weighing | 1 | 0,00 | 0,24 | 0,03 |
| FURFURYL ALCOHOL | Weighing | 2,5 | 0,06 | 0,24 | 0,03 |
| FURFURYL ALCOHOL | Weighing | 2,5 | 0,06 | 0,24 | 0,03 |
| FURFURYL ALCOHOL | Weighing | 2,5 | 0,05 | 0,24 | 0,03 |
| FURFURYL ALCOHOL | Packaging | 0,25 | 0,11 | 0,12 | 0,002 |
| FURFURYL ALCOHOL | Packaging | 0,25 | 0,10 | 0,12 | 0,002 |
| FURFURYL ALCOHOL | Packaging | 1 | 0,32 | 0,48 | 0,003 |
| FURFURYL ALCOHOL | Packaging | 1 | 0,32 | 0,48 | 0,003 |
| FURFURYL ALCOHOL | Packaging | 2,5 | 0,32 | 0,48 | 0,01 |
| FURFURYL ALCOHOL | Packaging | 1 | 0,27 | 0,48 | 0,003 |
| FURFURYL ALCOHOL | Packaging | 0,25 | 0,04 | 0,12 | 0,003 |
| FURFURYL ALCOHOL | Packaging | 2,5 | 0,09 | 0,48 | 0,01 |
| FURFURYL ALCOHOL | Packaging | 2,5 | 0,06 | 0,48 | 0,01 |
| HEXANE | Transfering | 450 | 11 | 26 | 0,9 |
| HEXANE | Transfering | 120 | 0,50 | 5,2 | 0,9 |
| HEXANE | Transfering | 450 | 0,22 | 26 | 0,45 |
| HEXANE | Transfering | 150 | 3,3 | 5,2 | 0,9 |
| HEXANE | Transfering | 150 | 1,5 | 5,2 | 0,45 |
| HEXANE | Transfering | 150 | 0,69 | 5,2 | 0,45 |
| HEXANE | Transfering | 150 | 0,61 | 5,2 | 2,7 |
| HEXANE | Transfering | 150 | 16 | 36 | 15 |
| HEXANE | Transfering | 150 | 6,1 | 26 | 0,45 |
| HEXANE | Transfering | 150 | 1,6 | 5,2 | 0,45 |
| ISOAMYL ACETATE | Weighing | 1 | 0,16 | 0,64 | 0,15 |
| ISOAMYL ACETATE | Weighing | 1 | 0,30 | 1,8 | 0,15 |
| ISOAMYL ACETATE | Weighing | 1 | 0,06 | 0,64 | 0,15 |
| ISOAMYL ACETATE | Weighing | 1 | 0,15 | 1,8 | 0,15 |
| ISOAMYL ACETATE | Weighing | 1 | 0,15 | 1,8 | 0,15 |
| ISOAMYL ACETATE | Weighing | 1 | 0,04 | 0,64 | 0,15 |
| ISOAMYL ALCOHOL | Weighing | 1 | 0,30 | 0,58 | 0,15 |
| ISOAMYL ALCOHOL | Weighing | 1 | 0,07 | 0,58 | 0,15 |
| ISOAMYL ALCOHOL | Weighing | 1 | 0,03 | 0,58 | 0,15 |
| ISOAMYL ALCOHOL | Weighing | 0,03 | 0,55 | 0,58 | 0,15 |
| ISOAMYL ALCOHOL | Weighing | 0,03 | 0,48 | 0,58 | 0,15 |
| ISOAMYL ALCOHOL | Weighing | 3 | 0,46 | 0,58 | 0,15 |
| ISOAMYL ALCOHOL | Weighing | 0,15 | 0,42 | 0,58 | 0,15 |
| ISOAMYL ALCOHOL | Weighing | 0,015 | 0,3 | 0,58 | 0,15 |
| ISOAMYL ALCOHOL | Weighing | 0,15 | 0,29 | 0,58 | 0,15 |
| ISOBUTYL ALCOHOL | Weighing | 430 | 1,1 | 15 | 30 |
| ISOBUTYL ALCOHOL | Packaging | 430,08 | 2,8 | 17 | 18 |
| ISOBUTYL ALCOHOL | Weighing | 0,08 | 0,01 | 0,90 | 0,90 |
| ISOBUTYL ALCOHOL | Weighing | 122 | 6,1 | 15 | 30 |
| ISOBUTYL ALCOHOL | Packaging | 122 | 0,87 | 1,2 | 1,0 |
| ISOBUTYL ALCOHOL | Weighing | 123,39 | 1,0 | 15 | 30 |
| ISOBUTYL ALCOHOL | Packaging | 123,39 | 0,11 | 0,36 | 0,03 |
| ISOBUTYL ALCOHOL | Weighing | 0,25 | 0,23 | 0,90 | 0,30 |
| ISOBUTYL ALCOHOL | Weighing | 0,25 | 0,02 | 0,90 | 0,30 |
| ISOBUTYL ALCOHOL | Weighing | 0,25 | 0,01 | 0,90 | 0,30 |
| ISOBUTYL ALCOHOL | Weighing | 1 | 0,18 | 0,90 | 0,30 |
| ISOBUTYL ALCOHOL | Weighing | 1 | 0,11 | 0,90 | 0,30 |
| ISOBUTYL ALCOHOL | Weighing | 1 | 0,03 | 0,90 | 0,30 |
| ISOBUTYL ALCOHOL | Weighing | 2,5 | 0,28 | 0,90 | 0,30 |
| ISOBUTYL ALCOHOL | Weighing | 2,5 | 0,17 | 0,90 | 0,30 |
| ISOBUTYL ALCOHOL | Weighing | 2,5 | 0,07 | 0,90 | 0,30 |
| ISOBUTYL ALCOHOL | Packaging | 1 | 1,4 | 1,8 | 0,03 |
| ISOBUTYL ALCOHOL | Packaging | 0,25 | 0,14 | 0,45 | 0,02 |
| ISOBUTYL ALCOHOL | Packaging | 1 | 0,43 | 1,8 | 0,03 |
| ISOBUTYL ALCOHOL | Packaging | 0,25 | 0,09 | 0,45 | 0,02 |
| ISOBUTYL ALCOHOL | Packaging | 0,25 | 0,09 | 0,45 | 0,03 |
| ISOBUTYL ALCOHOL | Packaging | 1 | 0,27 | 1,8 | 0,03 |
| ISOBUTYL ALCOHOL | Packaging | 2,5 | 0,26 | 1,8 | 0,09 |
| ISOBUTYL ALCOHOL | Packaging | 2,5 | 0,19 | 1,8 | 0,09 |
| ISOBUTYL ALCOHOL | Weighing | 0,015 | 0,96 | 0,90 | 0,15 |
| ISOBUTYL ALCOHOL | Weighing | 0,15 | 0,81 | 0,90 | 0,15 |
| ISOBUTYL ALCOHOL | Weighing | 0,15 | 0,77 | 0,90 | 0,15 |
| ISOBUTYL ALCOHOL | Weighing | 0,03 | 0,71 | 0,90 | 0,15 |
| ISOBUTYL ALCOHOL | Weighing | 0,03 | 0,5 | 0,90 | 0,15 |
| ISOBUTYL ALCOHOL | Weighing | 0,015 | 0,43 | 4,5 | 0,3 |
| ISOPROPYL ALCOHOL | Weighing | 0,15 | 2,2 | 2,2 | 0,015 |
| ISOVALERIC ALDEHYDE | Weighing | 0,25 | 0,15 | 2,8 | 0,30 |
| ISOVALERIC ALDEHYDE | Weighing | 0,25 | 0,04 | 2,8 | 0,30 |
| ISOVALERIC ALDEHYDE | Weighing | 0,25 | 0,02 | 2,8 | 0,30 |
| ISOVALERIC ALDEHYDE | Weighing | 1 | 0,13 | 2,8 | 0,30 |
| ISOVALERIC ALDEHYDE | Weighing | 1 | 0,08 | 2,8 | 0,30 |
| ISOVALERIC ALDEHYDE | Weighing | 1 | 0,00 | 2,8 | 0,30 |
| ISOVALERIC ALDEHYDE | Weighing | 2,5 | 0,42 | 2,8 | 0,30 |
| ISOVALERIC ALDEHYDE | Weighing | 2,5 | 0,17 | 2,8 | 0,30 |
| ISOVALERIC ALDEHYDE | Weighing | 2,5 | 0,15 | 2,8 | 0,30 |
| ISOVALERIC ALDEHYDE | Packaging | 2,5 | 6,9 | 11 | 0,09 |
| ISOVALERIC ALDEHYDE | Packaging | 1 | 1,3 | 5,6 | 0,03 |
| ISOVALERIC ALDEHYDE | Packaging | 2,5 | 0,99 | 5,6 | 0,09 |
| ISOVALERIC ALDEHYDE | Packaging | 2,5 | 0,83 | 5,6 | 0,09 |
| ISOVALERIC ALDEHYDE | Packaging | 1 | 0,78 | 5,6 | 0,03 |
| ISOVALERIC ALDEHYDE | Packaging | 0,25 | 0,18 | 1,4 | 0,02 |
| ISOVALERIC ALDEHYDE | Packaging | 0,25 | 0,12 | 1,4 | 0,02 |
| ISOVALERIC ALDEHYDE | Packaging | 1 | 0,48 | 5,6 | 0,03 |
| ISOVALERIC ALDEHYDE | Packaging | 0,25 | 0,08 | 1,4 | 0,03 |
| METHANOL | Transfering | 150 | 1 | 33 | 1,4 |
| METHANOL | Transfering | 900 | 3 | 24 | 0,9 |
| METHANOL | Transfering | 3,7 | 8 | 79 | 30 |
| METHANOL | Transfering | 3,2 | 4 | 16 | 15 |
| METHANOL | Transfering | 144 | 3 | 16 | 15 |
| METHANOL | Transfering | 900 | 2 | 24 | 0,9 |
| METHANOL | Transfering | 6 | 1 | 16 | 15 |
| METHANOL | Transfering | 150 | 3 | 5 | 0,45 |
| METHANOL | Transfering | 150 | 5 | 5 | 0,45 |
| METHANOL | Transfering | 150 | 4 | 5 | 0,45 |
| METHANOL | Transfering | 150 | 3 | 5 | 0,45 |
| PROPIONIC ALDEHYDE | Weighing | 0,13 | 0,03 | 7,3 | 0,90 |
| PROPIONIC ALDEHYDE | Weighing | 0,25 | 0,47 | 7,3 | 0,90 |
| PROPIONIC ALDEHYDE | Weighing | 0,25 | 0,37 | 7,3 | 0,90 |
| PROPIONIC ALDEHYDE | Weighing | 0,25 | 0,19 | 7,3 | 0,90 |
| PROPIONIC ALDEHYDE | Weighing | 1 | 0,67 | 7,3 | 0,90 |
| PROPIONIC ALDEHYDE | Weighing | 1 | 0,28 | 7,3 | 0,90 |
| PROPIONIC ALDEHYDE | Weighing | 1 | 0,01 | 7,3 | 0,90 |
| PROPIONIC ALDEHYDE | Weighing | 2,5 | 1,4 | 7,3 | 0,90 |
| PROPIONIC ALDEHYDE | Weighing | 2,5 | 0,40 | 7,3 | 0,90 |
| PROPIONIC ALDEHYDE | Weighing | 2,5 | 0,22 | 7,3 | 0,90 |
| PROPIONIC ALDEHYDE | Packaging | 2,5 | 7,2 | 15 | 0,27 |
| PROPIONIC ALDEHYDE | Packaging | 1 | 3,0 | 15 | 0,09 |
| PROPIONIC ALDEHYDE | Packaging | 2,5 | 2,9 | 15 | 0,27 |
| PROPIONIC ALDEHYDE | Packaging | 2,5 | 2,8 | 15 | 0,27 |
| PROPIONIC ALDEHYDE | Packaging | 1 | 2,1 | 15 | 0,09 |
| PROPIONIC ALDEHYDE | Packaging | 0,25 | 0,51 | 3,7 | 0,05 |
| PROPIONIC ALDEHYDE | Packaging | 0,25 | 0,45 | 3,7 | 0,09 |
| PROPIONIC ALDEHYDE | Packaging | 0,25 | 0,44 | 3,7 | 0,05 |
| PROPIONIC ALDEHYDE | Packaging | 1 | 1,5 | 15 | 0,09 |
| PROPYL ALCOHOL | Weighing | 74 | 0,19 | 4,0 | 30 |
| PROPYL ALCOHOL | Packaging | 74 | 0,07 | 0,12 | 0,02 |
| PROPYL ALCOHOL | Weighing | 0,25 | 0,33 | 1,2 | 0,30 |
| PROPYL ALCOHOL | Weighing | 0,25 | 0,10 | 1,2 | 0,30 |
| PROPYL ALCOHOL | Weighing | 0,25 | 0,05 | 1,2 | 0,30 |
| PROPYL ALCOHOL | Weighing | 1 | 0,55 | 1,2 | 0,30 |
| PROPYL ALCOHOL | Weighing | 1 | 0,19 | 1,2 | 0,30 |
| PROPYL ALCOHOL | Weighing | 1 | 0,11 | 1,2 | 0,30 |
| PROPYL ALCOHOL | Weighing | 2,5 | 0,36 | 1,2 | 0,30 |
| PROPYL ALCOHOL | Weighing | 2,5 | 0,15 | 1,2 | 0,30 |
| PROPYL ALCOHOL | Weighing | 2,5 | 0,15 | 1,2 | 0,30 |
| PROPYL ALCOHOL | Packaging | 2,5 | 4,8 | 4,8 | 0,09 |
| PROPYL ALCOHOL | Packaging | 0,25 | 0,28 | 0,61 | 0,02 |
| PROPYL ALCOHOL | Packaging | 1 | 0,96 | 2,4 | 0,03 |
| PROPYL ALCOHOL | Packaging | 0,25 | 0,23 | 0,61 | 0,02 |
| PROPYL ALCOHOL | Packaging | 0,25 | 0,17 | 0,61 | 0,03 |
| PROPYL ALCOHOL | Packaging | 1 | 0,64 | 2,4 | 0,03 |
| PROPYL ALCOHOL | Packaging | 2,5 | 0,60 | 2,4 | 0,09 |
| PROPYL ALCOHOL | Packaging | 2,5 | 0,45 | 2,4 | 0,09 |
| PROPYL ALCOHOL | Weighing | 0,15 | 1,3 | 1,2 | 0,15 |
| PROPYL ALCOHOL | Weighing | 0,15 | 1,2 | 1,2 | 0,15 |
| PROPYL ALCOHOL | Weighing | 0,015 | 1,2 | 1,2 | 0,15 |
| PROPYL ALCOHOL | Weighing | 0,03 | 0,97 | 1,2 | 0,15 |
